# The PERK/ATF4 pathway is required for metabolic reprogramming and progressive lung fibrosis

**DOI:** 10.1172/jci.insight.189330

**Published:** 2025-04-10

**Authors:** Jyotsana Pandey, Jennifer L. Larson-Casey, Mallikarjun H. Patil, Chao He, Nisarat Pinthong, A. Brent Carter

**Affiliations:** 1 Department of Medicine, University of Alabama at Birmingham, Birmingham, Alabama, USA.; 2 Baylor College of Medicine, Houston, Texas, USA.; 3 Birmingham Veterans Administration Medical Center, Birmingham, Alabama, USA.

**Keywords:** Immunology, Pulmonology, Fatty acid oxidation, Fibrosis, Macrophages

## Abstract

Asbestosis is a prototypical type of fibrosis that is progressive and does not resolve. ER stress is increased in multiple cell types that contribute to fibrosis; however, the mechanism(s) by which ER stress in lung macrophages contributes to fibrosis is poorly understood. Here, we show that ER stress resulted in protein kinase RNA-like ER kinase (PERK; *Eif2ak3*) activation in humans with asbestosis. Similar results were seen in asbestos-injured mice. Mice harboring a conditional deletion of *Eif2ak3* were protected from fibrosis. Lung macrophages from asbestosis individuals had evidence of metabolic reprogramming to fatty acid oxidation (FAO). *Eif2ak3^fl/fl^* mice had increased oxygen consumption rate (OCR), whereas OCR in *Eif2ak3^–/–^* *Lyz2-cre* mice was reduced to control levels. PERK increased activating transcription factor 4 (*Atf4*) expression, and ATF4 bound to the *Ppargc1a* promoter to increase its expression. GSK2656157, a PERK-specific inhibitor, reduced FAO, *Ppargc1a*, and *Aft4* in lung macrophages and reversed established fibrosis in mice. These observations suggest that PERK is a therapeutic target to reverse established fibrosis.

## Abstract 2

Macrophages drive fibrosis development and their metabolism contributes to this process. Stress in the endoplasmic reticulum promotes metabolic reprogramming the mediates fibrosis progression.

## Graphical abstract

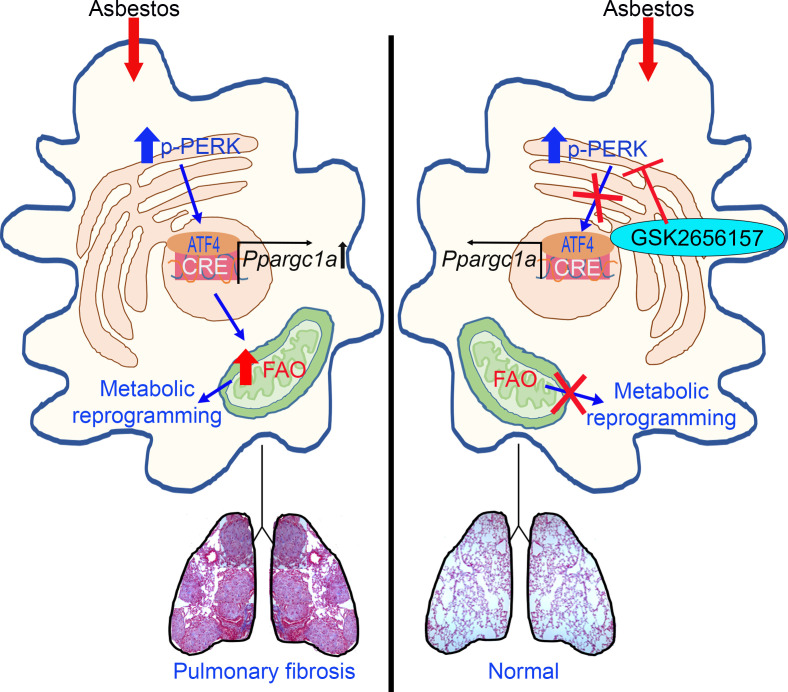

## Introduction

Asbestos-induced pulmonary fibrosis is a progressive, nonresolving disease that results in 40,000 deaths annually in the United States (1). Recently approved antifibrotic therapies have limited efficacy and do not improve quality of life or alter mortality (2, 3). Although strict regulatory measures are in place, more than 1.3 million workers in the United States are exposed to hazardous levels of asbestos annually.

Lung macrophages play a critical role in mediating asbestos-induced fibrosis by initiating an immune response. Macrophages contribute importantly in the immune response to both infectious and noninfectious agents, but they are also critical in the repair responses to tissue injury (4). These diverse functions are dependent on their ability to modify into phenotypically distinct subpopulations. Macrophages develop mixed phenotypes in complex pathological conditions, such as injury and fibrosis, and polarize to a predominant cellular and metabolic phenotype depending on the duration and stage of injury or repair (5–9).

Typically, monocytes contribute to tissue repair by being recruited to injured areas in response to CCL2 secretion from resident alveolar macrophages (RAMs) (10). RAMs are critical in the clearance of cellular debris and essential for resolving lung inflammation and injury (11). However, in tissues under continuous injury, recruitment can be detrimental. Bone marrow–derived monocytes have the capacity to migrate into tissues and differentiate into tissue-resident macrophages (12). Monocyte-derived macrophages (MDMs) drive fibrotic lung remodeling and mediate disease progression through the expression and secretion of pro-fibrotic mediators (13–15). Asbestos-induced fibrosis is nonresolving, as the fibers persist in the lung. If regeneration is not permissible, fibrosis occurs. Moreover, MDMs are resistant to apoptosis and are a critical source of fibrotic growth factors (15–18). Targeting these cells to disrupt fibrotic progression in asbestos-induced fibrosis may provide an effective strategy for therapeutic intervention.

Macrophages display metabolic heterogeneity, and distinct metabolic profiles exist between tissue-resident macrophages and recruited macrophages (19, 20). Highly dependent on oxidative phosphorylation (OXPHOS) and fatty acid metabolism for energy, tissue-resident macrophages (Kupffer cells) play a crucial role in resolution of liver fibrosis (21). However, infiltrating hepatic macrophages that exhibit increased glycolytic activity and lactate production promote liver fibrosis. Similar macrophage metabolic profiles are seen in cardiac and kidney fibrosis (19, 22). Metabolic reprogramming in lung macrophages is known to be present during lung remodeling; however, the mechanism by which this occurs is not entirely clear. Peroxisome proliferator–activated receptor γ coactivator-1α (PGC-1α; *Ppargc1a*) increases the enzymatic capacity for OXPHOS and fatty acid oxidation (FAO), and it is increased in macrophages during fibrosis (14, 23, 24). Although its transcriptional induction has many points of regulation, the modulation of *Ppargc1a* transcription in macrophages in asbestosis or established fibrosis has not been determined.

ER stress occurs in several diseases, including aging, cancer, and fibrosis. Prolonged ER stress and unfolded protein response (UPR) activates glucose-related peptide 78, GRP78 (BiP), with further activation of protein kinase RNA-like ER kinase (PERK), inositol-requiring enzyme-1α (IRE1α), and activating transcription factor 6 (ATF6) signaling, which have specific functions in regulating protein synthesis, protein folding, and degradation of misfolded proteins. ER stress is well characterized in several lung cell types involved in fibrosis. ER stress generates inflammatory cytokines in alveolar epithelial cells (AECs) and can induce apoptosis (25–27). It mediates fibroblast differentiation in response to TGF-β1 exposure (28, 29). Although ER stress has been linked to alternative activation of macrophages in foam cells in atherosclerosis and in adipose tissue macrophages in obesity (30, 31), its role in lung macrophages in established fibrosis is poorly understood.

## Results

### Asbestos activates PERK in lung macrophages.

**Figure 1:**
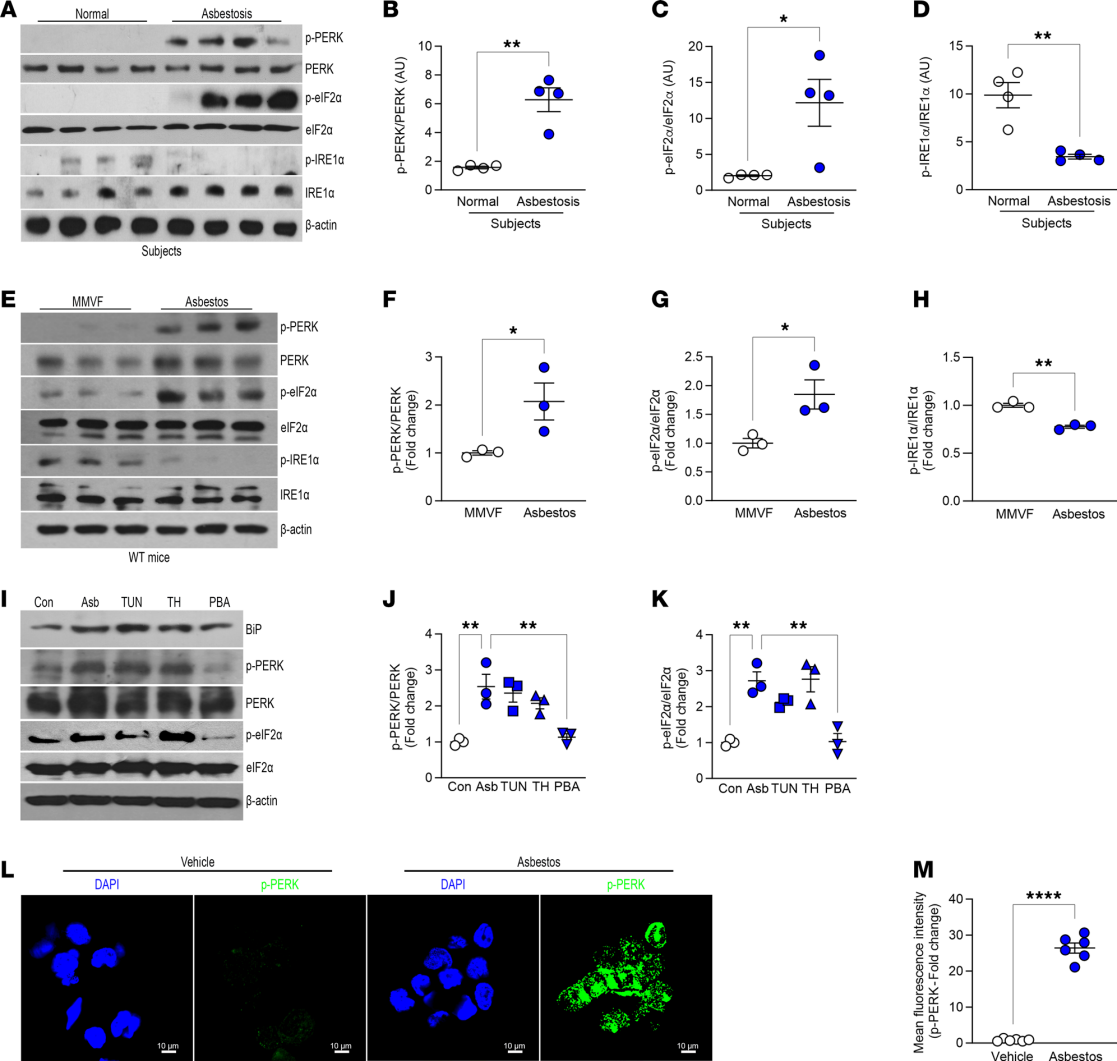
Asbestos activates PERK in lung macrophages. (**A**) Lung macrophages from normal and asbestosis humans were obtained by bronchoalveolar lavage (BAL) and subjected to immunoblot analysis (*n* = 4). Densitometry of (**B**) phosphorylated (p-) PERK, (**C**) p-eIF2α, and (**D**) p-IRE1α in humans. (**E**) WT mice were exposed to man-made vitreous fiber (MMVF) or asbestos (100 μg intratracheally; i.t.). BAL was performed on day 21, and lung macrophages were subjected to immunoblot analysis (*n* = 3). Densitometry of (**F**) p-PERK, (**G**) p-eIF2α, and (**H**) p-IRE1α from exposed mice. (**I**) Macrophages were exposed to vehicle (Con), asbestos (Asb), tunicamycin (TUN), thapsigargin (TH), or 4PBA (PBA) and subjected to immunoblot analysis. Densitometry of (**J**) p-PERK and (**K**) p-eIF2α (*n* = 3). (**L**) Macrophages were exposed to vehicle or asbestos and stained for p-PERK. The staining was imaged by confocal microscopy, scale bars at 10 μm and 40×. (**M**) Quantification of mean fluorescence intensity (*n* = 3). Data shown as mean ± SEM. Two-tailed Student’s *t* test in **B**–**D**, **F**–**H**, and **M**. One-way ANOVA with Tukey’s post hoc comparison in **J** and **K**. *P ≤ 0.05, **P ≤ 0.01, and ****P ≤ 0.0001.

ER stress is well described in AECs (25) and fibroblasts (28, 29). ER stress and activation of UPR are associated with alternative (pro-fibrotic) activation of macrophages (30). Lung macrophages from humans with asbestosis had increased PERK activation and increased phosphorylation of eukaryotic translation initiation factor 2α (eIF2α) that was absent in healthy humans (Figure 1, A–C). In contrast, IRE1α phosphorylation was absent in asbestosis participants (Figure 1, A and D), suggesting selective activation of PERK in humans with asbestos-induced fibrosis. Similar findings were seen in lung macrophages from asbestos-injured mice (Figure 1, E–H).

To further verify our observations, we exposed macrophages to asbestos or ER stress activators, tunicamycin (TUN) and thapsigargin (TH), or an inhibitor (4PBA). BiP was increased with asbestos, TUN, and TH and unchanged in cells treated with 4PBA (Figure 1I). Asbestos significantly increased PERK phosphorylation similar to the levels seen with TUN and TH, whereas 4PBA significantly inhibited PERK activation (Figure 1, I and J). eIF2α had a similar activation profile as PERK (Figure 1, I–K). The effect of asbestos-induced PERK activation was further verified by confocal microscopy (Figure 1, L and M). Macrophages exposed to asbestos had 25-fold greater PERK phosphorylation compared with the vehicle control.

### PERK is required for asbestos-induced lung fibrosis.

**Figure 2:**
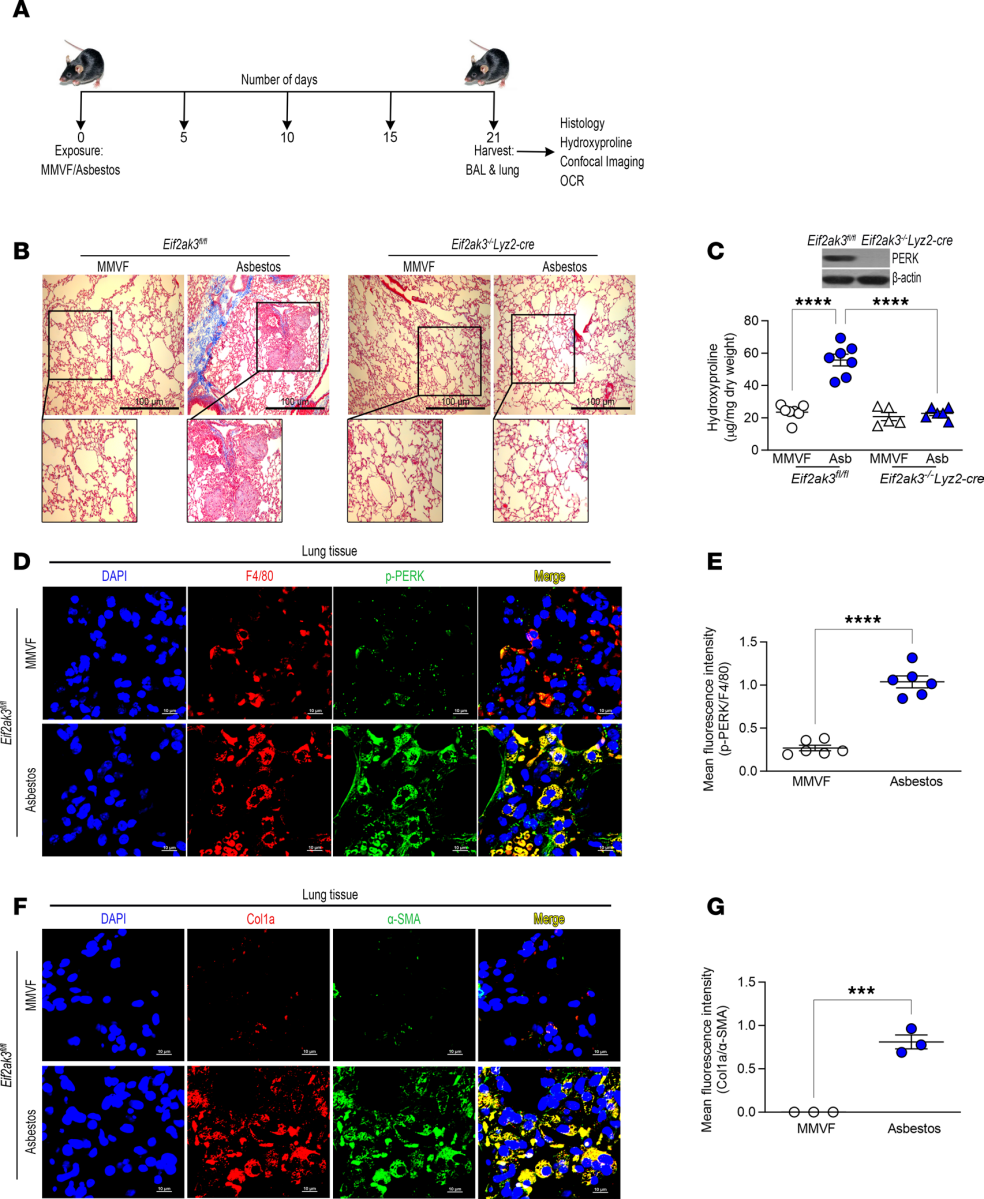
PERK is required for asbestos-induced lung fibrosis. (**A**) Schematic representation for animal study: *Eif2ak3^fl/fl^* and *Eif2ak3^–/–^* *Lyz2-cre* littermates were exposed to MMVF or asbestos. Lung macrophages and lung tissues were isolated at 21 days and subjected to histology, hydroxyproline, and confocal imaging. OCR, oxygen consumption rate. (**B**) Masson’s trichome staining, scale bars at 100 μm and 10×. (**C**) Hydroxyproline assay (*n* = 5–7). Inset, immunoblot analysis of PERK in BAL cells from *Eif2ak3^fl/fl^* and *Eif2ak3^–/–^* *Lyz2-cre* mice. (**D**) Lung tissue sections from MMVF- or asbestos-injured mice were stained with p-PERK and F4/80. The staining was imaged by confocal microscopy, scale bars at 10 μm and 40×. (**E**) Quantification of mean fluorescence intensity (*n* = 6). (**F**) Lung tissue sections were stained with Col1a and α-SMA and imaged by confocal microscopy, scale bars at 10 μm and 40×. (**G**) Quantification of mean fluorescence intensity (*n* = 3). Data shown as mean ± SEM. One-way ANOVA with Tukey’s post hoc comparison in **C**. Two-tailed Student’s *t* test in **E** and **G**. ***P ≤ 0.001, ****P ≤ 0.0001. (See also Supplemental Figure 1.)

PERK regulates protein synthesis and supports cell survival (32). To test the effect of PERK activation in fibrotic lung macrophages, we exposed *Eif2ak3^fl/fl^* mice and mice harboring a conditional deletion of *Eif2ak3* in macrophages (*Eif2ak3^–/–^* *Lyz2-cre*) to asbestos (Figure 2A). Asbestos-injured *Eif2ak3^fl/fl^* mice showed architectural distortion with parenchymal fibrosis, increased collagen deposition, and fibroblastic foci, whereas MMVF-exposed mice showed normal lung structure (Figure 2B). Asbestos-exposed *Eif2ak3^–/–^* *Lyz2-cre* mice were protected from lung fibrosis (Figure 2C). PERK was absent in lung macrophages but not in AECs (Figure 2C and Supplemental Figure 1A; supplemental material available online with this article; https://doi.org/10.1172/jci.insight.189330DS1). The histological results were verified biochemically by hydroxyproline assay (Figure 2C). Lung tissue from asbestos-injured *Eif2ak3^fl/fl^* mice had significantly increased PERK phosphorylation in macrophages compared with MMVF-exposed control mice (Figure 2, D and E). PERK phosphorylation was also seen in type II AECs (Supplemental Figure 1B). Moreover, asbestos exposure mediated a significant increase in collagen type 1α (Col1a) and α–smooth muscle actin (α-SMA) compared with lungs from MMVF-exposed mice (Figure 2, F and G). These data demonstrate that activation of PERK in macrophages is required for asbestos-induced lung fibrosis.

### PERK activation increases in a time-dependent manner in BAL cells.

**Figure 3:**
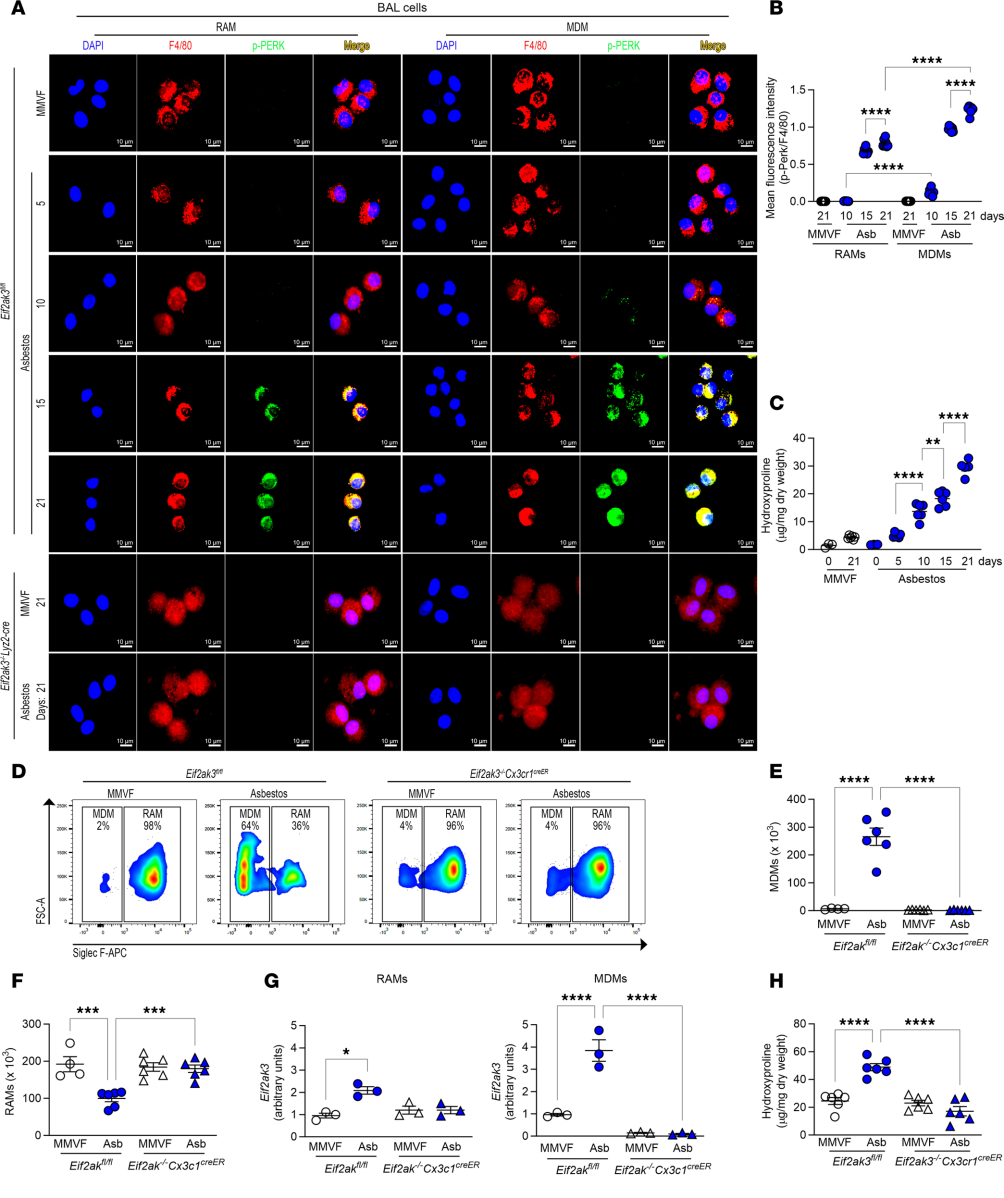
PERK activation increases in a time-dependent manner in BAL cells. (**A**) Lung macrophages were obtained by BAL from *Eif2ak3^fl/fl^* and *Eif2ak3^–/–^* *Lyz2-cre* littermates from MMVF- or asbestos-injured mice. RAMs and MDMs were FACS-sorted on days 0, 5, 10, 15, and 21 and stained for p-PERK and F4/80. The staining was imaged by confocal microscopy, scale bars at 10 μm and 40×. (**B**) Quantification of mean fluorescence intensity (*n* = 7). (**C**) Hydroxyproline assay in lung tissues at indicated time points (*n* = 3–6). *Eif2ak3^fl/fl^* and *Eif2ak3^–/–^* *Cx3cr1^creER^* mice were administered tamoxifen and exposed to MMVF or asbestos. BAL was performed 21 days after exposure. (**D**) Representative flow plots with percentages and number of (**E**) MDMs (*n* = 6) and (**F**) RAMs (*n* = 4–6). FSC, forward scatter. (**G**) *Eif2ak3* expression in FACS-sorted BAL cells (*n* = 3). (**H**) Hydroxyproline analysis in lung tissues (*n* = 6). Data shown as mean ± SEM. One-way ANOVA with Tukey’s post hoc comparison. *P ≤ 0.05, **P ≤ 0.01, ***P ≤ 0.001, and ****P ≤ 0.0001. (See also Supplemental Figure 2.)

MDMs play an essential role in fibrotic development and progression. Previous data showed that asbestos-induced lung fibrosis started 10 days after exposure in mice and continued to progress without resolution (7). We determined if PERK activation correlated with fibrosis progression. Asbestos-injured *Eif2ak3^fl/fl^* mice expressed p-PERK in MDMs at day 10, and p-PERK continued to increase in a time-dependent manner through day 21 (Figure 3A and Supplemental Figure 2A). PERK phosphorylation was absent in RAMs at day 5 and day 10 and appeared at day 15 and day 21 compared with the MMVF control. There was significantly less p-PERK in RAMs compared with MDMs from asbestos-injured mice at days 10, 15, and 21 (Figure 3B). RAMs and MDMs from *Eif2ak3^–/–^* *Lyz2-cre* mice showed an absence of PERK phosphorylation. Furthermore, the hydroxyproline in *Eif2ak3^fl/fl^* mice mirrored the activation of PERK in MDMs by increasing in a time-dependent manner starting at day 10 (Figure 3C).

Previous data from our group and others demonstrated that asbestos and bleomycin significantly increased the recruitment of MDMs in the lung (13, 15, 17, 24, 33); however, the role of PERK in MDM recruitment is not known. Using a preventive model, we administered tamoxifen to mice and subsequently exposed them to MMVF or asbestos. BAL cells from *Eif2ak3^fl/fl^* and mice harboring conditional deletion of *Eif2ak3* in macrophages (*Eif2ak3^–/–^* *Cx3cr1^creER^*) were subjected to FACS (Supplemental Figure 2A). The number of MDMs was significantly increased in asbestos-injured *Eif2ak3^fl/fl^* mice, whereas there was no increase in the *Eif2ak3^–/–^* *Cx3cr1^creER^* mice (Figure 3, D and E). Asbestos exposure reduced RAM numbers in the *Eif2ak3^fl/fl^* mice, and there was no change in the number of RAMs in the *Eif2ak3^–/–^* *Cx3cr1^creER^* mice compared with the MMVF control (Figure 3, D and F). The absence of PERK in MDMs from *Eif2ak3^–/–^* *Cx3cr1^creER^* mice (Figure 3G) inhibited fibrotic remodeling (Figure 3H).

CCL2 levels in BAL fluid from asbestos-injured *Eif2ak3^fl/fl^* mice were significantly greater than in *Eif2ak3^–/–^* *Lyz2-cre* mice on day 21 (Supplemental Figure 2B). To understand the impact of PERK on macrophage recruitment, we subjected BAL cells to FACS 7 days after asbestos exposure. While RAMs were prominent in both strains, a greater number of RAMs than MDMs was present in *Eif2ak3^–/–^* *Lyz2-cre* mice (Supplemental Figure 2C). The reduction in MDMs in asbestos-exposed *Eif2ak3^–/–^* *Lyz2-cre* mice was associated with markedly reduced *Ccl2* expression in FACS-sorted RAMs (Supplemental Figure 2D).

Asbestos-exposed *Eif2ak3^–/–^* *Lyz2-cre* mice administered clodronate liposomes 7 days after exposure showed similar numbers of RAMs and MDMs at 21 days (Supplemental Figure 2E). Differentiation of MDMs to RAMs was not observed, as the number of MDMs was similar in mice that received control or clodronate-loaded liposomes. Asbestos-injured *Eif2ak3^fl/fl^* mice showed increased number of annexin V^+^ RAMs compared with MMVF-exposed or *Eif2ak3^–/–^* *Cx3cr1^creER^* mice (Supplemental Figure 2F). No difference was observed in the number of annexin V^+^ MDMs from either strain of mice (Supplemental Figure 2G), suggesting that PERK does not influence MDM recruitment during the early inflammatory phase of lung injury but has a critical role during the later phase of fibrotic remodeling. These findings suggest ER stress, via increased activation of PERK in MDMs, was crucial for the development of asbestos-induced fibrosis, not the inflammatory recruitment.

### Asbestos-induced PERK activation mediates metabolic reprogramming.

**Figure 4:**
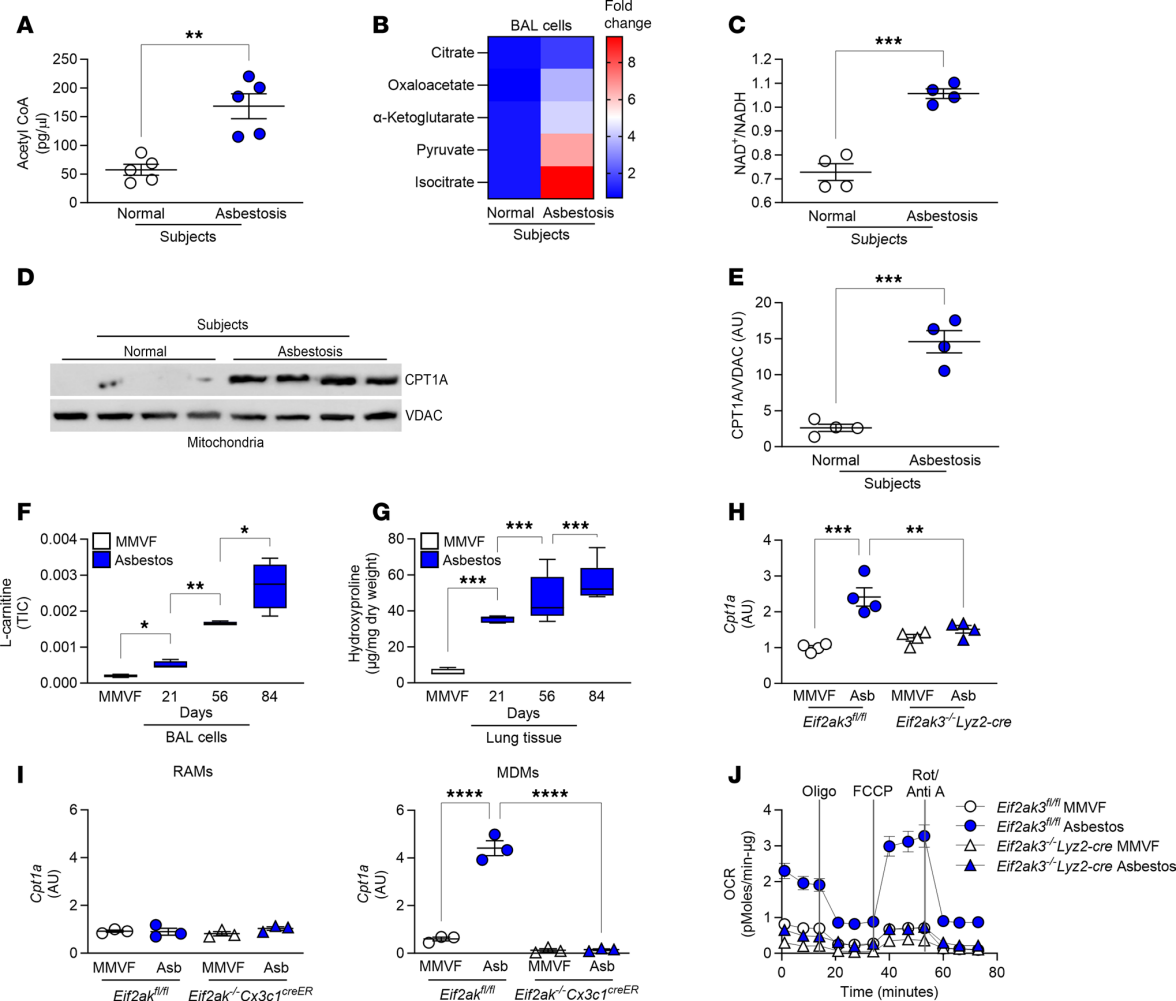
Asbestos-induced PERK activation mediates metabolic reprogramming. Lung macrophages were obtained by BAL from normal and asbestosis humans. (**A**) Acetyl-CoA concentration measured by fluorometry in humans (*n* = 5). (**B**) TCA metabolites measured by mass spectrometry in humans and shown as fold-change (*n* = 4–5). (**C**) Ratio of NAD^+^/NADH in humans measured by fluorometry (*n* = 4). (**D**) Immunoblot analysis of CPT1A and (**E**) densitometry of CPT1A in BAL cell mitochondrial fractions from humans (*n* = 4). WT mice were exposed to MMVF or asbestos (100 μg i.t.). VDAC, voltage-dependent anion channel. (**F**) Lung macrophages from exposed mice were subjected to mass spectrometry. l-carnitine was normalized to total ion chromatography (TIC) (*n* = 3–4). (**G**) Hydroxyproline assay in lung tissues at the designated time points (*n* = 5). *Cpt1a* mRNA expression in (**H**) BAL cells isolated at day 21 from exposed *Eif2ak3^fl/fl^* and *Eif2ak3^–/–^* *Lyz2-cre* mice (*n* = 4) and (**I**) FACS-sorted RAMs and MDMs isolated from BAL at day 21 from *Eif2ak3^fl/fl^* and *Eif2ak3^–/–^* *Cx3cr1^creER^* mice (*n* = 3). (**J**) OCR kinetics in BAL cells isolated at day 21 from exposed *Eif2ak3^fl/fl^* and *Eif2ak3^–/–^* *Lyz2-cre* mice (*n* = 3). Oligo, oligomycin; FCCP, carbonyl cyanide *p*-trifluoromethoxyphenylhydrazone; Rot/Anti A, rotenone/antimycin A; min-μg, minute/μg protein. Data shown as mean ± SEM. Two-tailed Student’s *t* test in **A**, **C**, and **E**. One-way ANOVA with Tukey’s post hoc comparison in **F**–**I**. *P ≤ 0.05, **P ≤ 0.01, ***P ≤ 0.001, and ****P ≤ 0.0001. (See also Supplemental Figure 3.)

Emerging evidence suggests that loss of PERK impairs mitochondrial function (34), and PERK regulates mitochondrial activity to meet cellular metabolic demands (35). To understand the mechanism by which PERK mediates lung fibrosis progression, we examined metabolic components in human lung macrophages. Humans with asbestosis had significantly increased levels of acetyl-CoA in lung macrophages, the final product of FAO (Figure 4A). Asbestosis participants also had greater than 9-fold increase in isocitrate and other TCA cycle metabolites, including oxaloacetate (Figure 4B). The NAD^+^-to-NADH ratio in macrophages from asbestosis participants was also increased (Figure 4C). The oxidation of NADH into NAD^+^ at complex I provides 2 electrons for the electron transport chain, suggesting oxidation of NADH is augmented in asbestosis participants.

Previous data revealed that carnitine palmitoyltransferase 1A (CPT1A), the rate-limiting enzyme in FAO, was increased in lung macrophages from individuals with idiopathic pulmonary fibrosis (23). CPT1A was also significantly increased in asbestosis compared with healthy participants (Figure 4, D and E), suggesting metabolic reprogramming to FAO occurs in lung macrophages during asbestosis.

Asbestos-injured mice had a time-dependent increase in l-carnitine (Figure 4F), a substrate for FAO. This increase in l-carnitine correlated with a time-dependent increase in hydroxyproline during the 12 weeks after exposure (Figure 4G), indicating that metabolic reprogramming to FAO was maintained during progression of asbestos-induced fibrosis.

To determine the role of PERK in metabolic reprogramming in lung macrophages, we found the rate-limiting enzyme in FAO, *Cpt1a*, was significantly increased in *Eif2ak3^fl/fl^* mice, whereas mice harboring a conditional deletion of *Eif2ak3* in MDMs had a significant reduction in *Cpt1a* expression (Figure 4H). These results were verified by FACS. MDMs from asbestos-injured *Eif2ak3^fl/fl^* mice showed increased *Cpt1a* gene expression compared with MDMs from *Eif2ak3^–/–^* *Cx3cr1^creER^* mice, but no difference was detected in *Cpt1a* in RAMs from both strains (Figure 4I). Asbestos-injured *Eif2ak3^fl/fl^* mice had significantly increased OCR, whereas OCR was markedly decreased in the *Eif2ak3^–/–^* *Lyz2-cre* mice regardless of exposure to asbestos (Figure 4J and Supplemental Figure 3A). In contrast, overexpression of IRE1α^WT^ significantly reduced OCR near the level of the empty control (Supplemental Figure 3, B and C). These data strongly suggest the direct involvement of PERK in metabolic reprogramming to FAO in lung macrophages during fibrotic remodeling.

### PERK activates PGC-1α by increasing ATF4 in lung macrophages.

**Figure 5:**
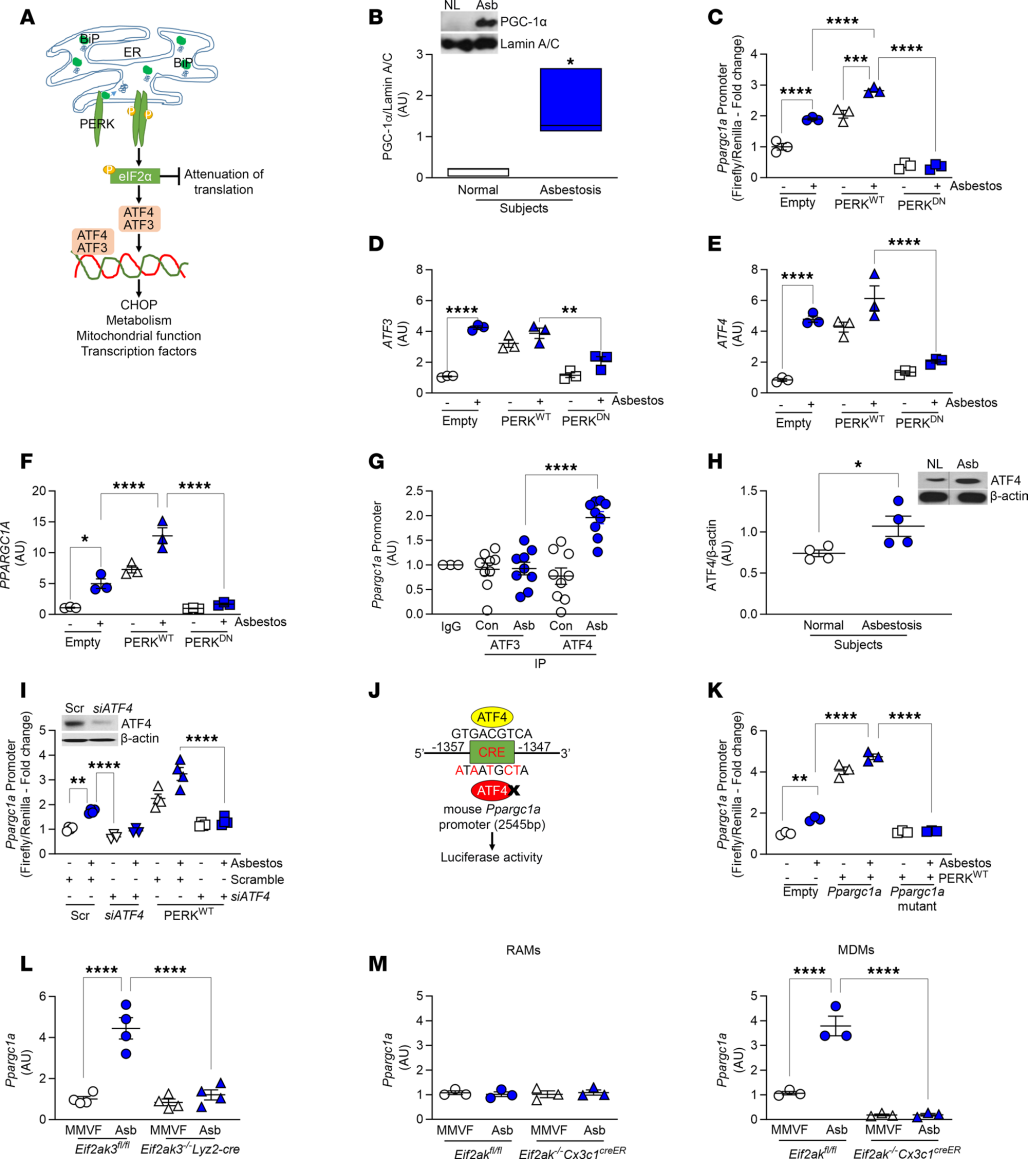
PERK activates PGC-1α by increasing ATF4 in lung macrophages. (**A**) Schematic representation of PERK pathway activation and downstream signaling molecules. CHOP, C/EBP homologous protein. (**B**) Lung macrophages from normal and asbestosis humans were obtained by BAL. Densitometry of immunoblot. Inset, immunoblot analysis of PGC-1α (*n* = 3). (**C**) Macrophages were cotransfected with renilla luciferase plasmid, pGL3-*Ppargc1a* luciferase promoter, and empty, PERK^WT^, or PERK^DN^ and exposed to asbestos (24 hours). *Ppargc1a* promoter activity was determined by measuring firefly and renilla luciferase (*n* = 3). (**D**) *ATF3* (*n* = 3), (**E**) *ATF4* (*n* = 3), and (**F**) *PPARGC1A* mRNA expression (*n* = 3) in transfected macrophages exposed to asbestos. (**G**) Macrophages were exposed to control or asbestos and subjected to ChIP with antibodies against ATF3 or ATF4 followed by real-time PCR to determine *Ppargc1a* promoter binding (*n* = 3–9). (**H**) Lung macrophages were obtained from normal and asbestosis humans by BAL. Densitometry of immunoblot. Inset, immunoblot analysis of ATF4 (*n* = 4). (**I**) Macrophages were cotransfected with pGL3-*Ppargc1a* luciferase promoter combined with scramble or *ATF4* siRNA, and empty or PERK^WT^, and exposed to asbestos. *Ppargc1a* promoter activity (*n* = 3–4). Inset, immunoblot analysis for ATF4. (**J**) Schematic illustration of ATF4 binding site on *Ppargc1a* promoter in the cAMP response element (CRE) domain and mutation sites on specific residues. (**K**) *Ppargc1a* promoter luciferase activity in macrophages transfected with empty, PERK^WT^, or pGL3-*Ppargc1a* luciferase mutant and exposed to asbestos (*n* = 3). (**L**) *Ppargc1a* mRNA expression in BAL isolated at day 21 from exposed *Eif2ak3^fl/fl^* and *Eif2ak3^–/–^* *Lyz2-cre* mice (*n* = 4). (**M**) *Ppargc1a* mRNA expression in FACS-sorted RAMs and MDMs isolated at day 21 from exposed *Eif2ak3^fl/fl^* and *Eif2ak3^–/–^* *Cx3cr1^creER^* mice (*n* = 3). Data shown as mean ± SEM. Two-tailed Student’s *t* test in **B** and **H**. One-way ANOVA with Tukey’s post hoc comparison in **C**–**G**, **I**, and **K**–**M**. *P ≤ 0.05, **P ≤ 0.01, ***P ≤ 0.001, and ****P ≤ 0.0001. (See also Supplemental Figure 4.)

PERK phosphorylates eIF2α, which inhibits global protein translation and induces ATF4 translocation to the nucleus to activate ATF3 (Figure 5A). ATF4 and ATF3 are transcription factors that, when activated, have different fates in the cell. To determine the mechanism by which PERK mediated metabolic reprogramming, we focused on PGC-1α. Humans with asbestosis had significantly greater nuclear expression of PGC-1α compared with healthy humans (Figure 5B). We determined if PERK regulated expression of PGC-1α. Overexpression of PERK^WT^ significantly increased *Ppargc1a* promoter–driven luciferase activity alone and was augmented with asbestos exposure (Figure 5C). The luciferase activity in cells expressing PERK^DN^ was below the empty control. PERK^WT^ also increased gene expression of *Atf3* and *Atf4*, and the expression was significantly reduced to empty control levels with PERK^DN^ (Figure 5, D and E). Similar results were found for gene expression of *Ppargc1a* (Figure 5F). *Cpt1a* gene expression was also increased with PERK^WT^ (Supplemental Figure 4A).

We questioned if ATF3 and ATF4 were involved in PERK-mediated expression of *Ppargc1a*. ATF3 and ATF4 have the same DNA binding consensus sequence (GTGACGTCA). ChIP with ATF3 and ATF4 antibodies showed increased binding of ATF4 to the *Ppargc1a* promoter (Figure 5G). ATF3 was no different from the IgG control. Asbestosis participants also had significantly increased expression of ATF4 in lung macrophages compared with the healthy participants (Figure 5H); however, there was no difference in CHOP expression in lung macrophages from mice exposed to asbestos compared with MMVF (Supplemental Figure 4B). Overexpression of PERK^WT^ increased *Ppargc1a* promoter activity, while this effect was abolished when *Atf4* was silenced (Figure 5I), verifying PERK-mediated increase in *Ppargc1a* expression requires ATF4 nuclear localization and DNA binding to the *Ppargc1a* promoter.

To further validate the binding of ATF4 on the *Ppargc1a* promoter, we generated a *Ppargc1a* promoter with the ATF4 binding sequence mutated. The vector had the following mutations: -1349CT, -1350TC, -1352CT, -1354GA, and -1356GA (Figure 5J). PERK^WT^ significantly increased *Ppargc1a* promoter activity, whereas PERK^WT^ had no effect on the mutant promoter (Figure 5K). Furthermore, silencing *PERK* in macrophages abrogated PGC-1α expression in the nucleus (Supplemental Figure 4, C–F). In vivo, macrophages from *Eif2ak3^fl/fl^* asbestos-exposed mice had increased *Ppargc1a* gene expression, and the expression in the *Eif2ak3^–/–^* *Lyz2-cre* mice was at the level of the MMVF control (Figure 5L). Moreover, BAL cells subjected to FACS showed *Ppargc1a* was increased in MDMs from asbestos-injured *Eif2ak3^fl/fl^* mice (Figure 5M). There was no difference of *Ppargc1a* expression in RAMs. These observations suggest that during asbestos-induced fibrotic remodeling, PERK increased *Ppargc1a* expression by augmenting ATF4 binding to the *Ppargc1a* promoter to mediate transcription and metabolic reprogramming of MDMs to FAO.

### Pharmacological inhibition of PERK reverses established lung fibrosis.

To determine if targeting PERK provided therapeutic potential to halt or reverse fibrotic remodeling, we used the PERK-specific inhibitor GSK2656157 (GSK) to validate that PERK is a potential therapeutic target. A dose response showed that 5 μM and above decreased expression of p-eIF2α (Supplemental Figure 5A). Similar results were seen in AECs (Supplemental Figure 5B) and fibroblasts (Supplemental Figure 5C). GSK did not induce apoptosis in any cell type (Supplemental Figure 5, D–F).

**Figure 6:**
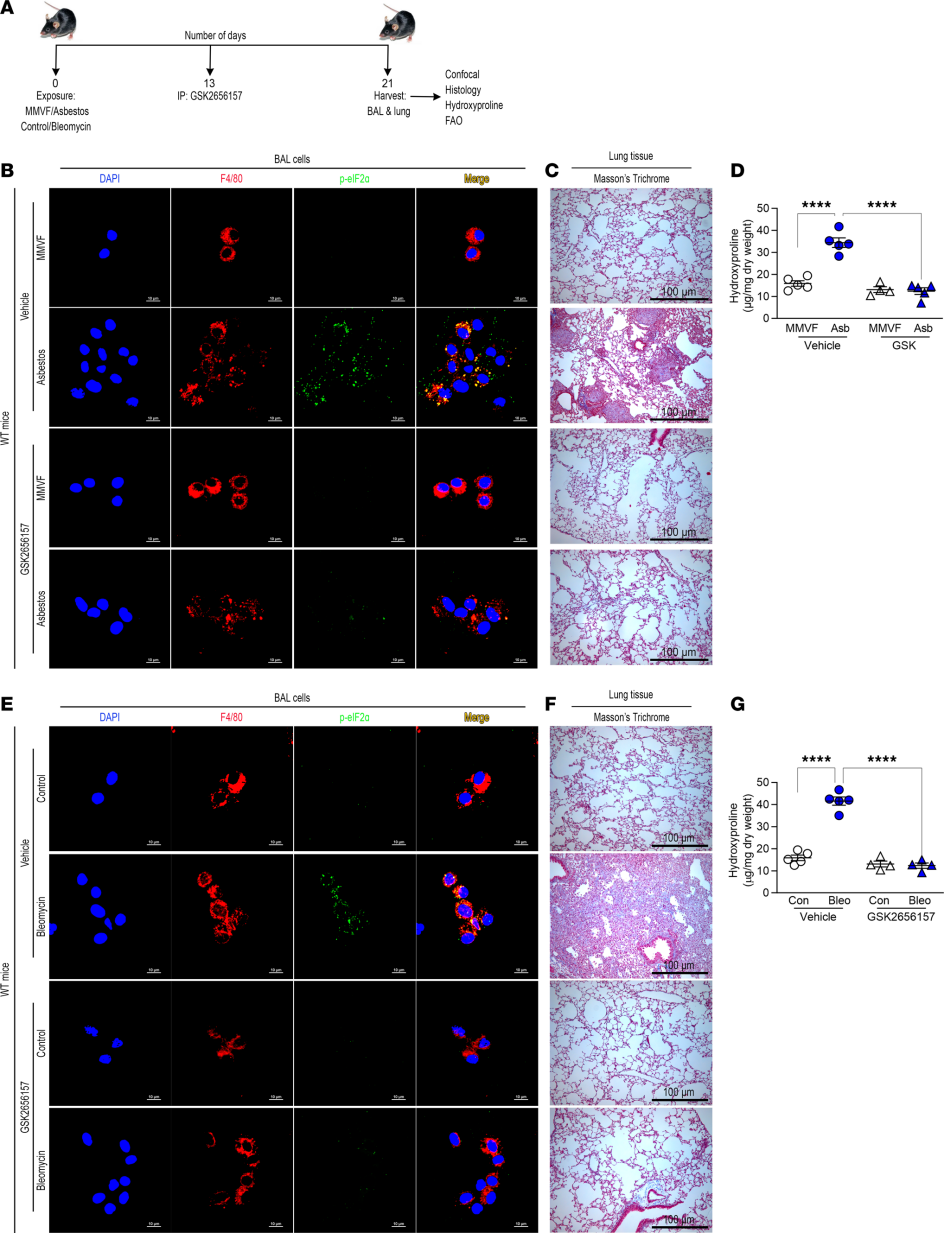
Pharmacological inhibition of PERK reverses established lung fibrosis. (**A**) Schematic of animal study. Thirteen days after exposure, GSK2656157 (GSK, 30 mg/kg i.p.) was administered daily to mice. (**B**) Lung macrophages were obtained by BAL from MMVF- or asbestos-exposed mice and subjected to staining for p-eIF2α and F4/80. The staining was imaged by confocal microscopy, scale bars at 10 μm or 40×. (**C**) Masson’s trichrome staining in representative micrographs from *n* = 4–5 mice per condition, scale bar at 100 μm and 10×. (**D**) Hydroxyproline assay in lung tissues (*n* = 4–5). (**E**) Lung macrophages were obtained by BAL from control or bleomycin-exposed mice and subjected to staining for p-eIF2α and F4/80, scale bars at 10 μm or 40×. (**F**) Masson’s trichrome staining in representative micrographs from (*n* = 4–5) mice per condition, scale bar at 100 μm and 10×. (**G**) Hydroxyproline assay in lung tissues (*n* = 4–5). Data shown as mean ± SEM. One-way ANOVA with Tukey’s post hoc comparison. ****P < 0.0001. (See also Supplemental Figure 5.)

Established fibrosis was present on day 13 in asbestos- or bleomycin-injured mice (Supplemental Figure 5, G–J). When fibrosis was established, we administered vehicle or GSK i.p. every day starting at day 13 (Figure 6A). Lung macrophages from asbestos-injured mice that received vehicle had increased eIF2α phosphorylation, whereas the macrophages from the mice treated with GSK had essentially no eIF2α phosphorylation (Figure 6B). The vehicle-treated mice exposed to asbestos had architectural distortion and widespread collagen deposition (Figure 6C). In contrast, the mice treated with GSK had complete resolution of established fibrosis. The histological changes were verified biochemically by hydroxyproline assay (Figure 6D).

Bleomycin-injured mice treated with GSK had no loss in body weight compared to bleomycin-injured mice that received vehicle (Supplemental Figure 6A). Bleomycin-injured mice replicated what was seen in the asbestos-injured mice with complete resolution of established fibrosis in mice treated with GSK (Figure 6, E–G).

**Figure 7:**
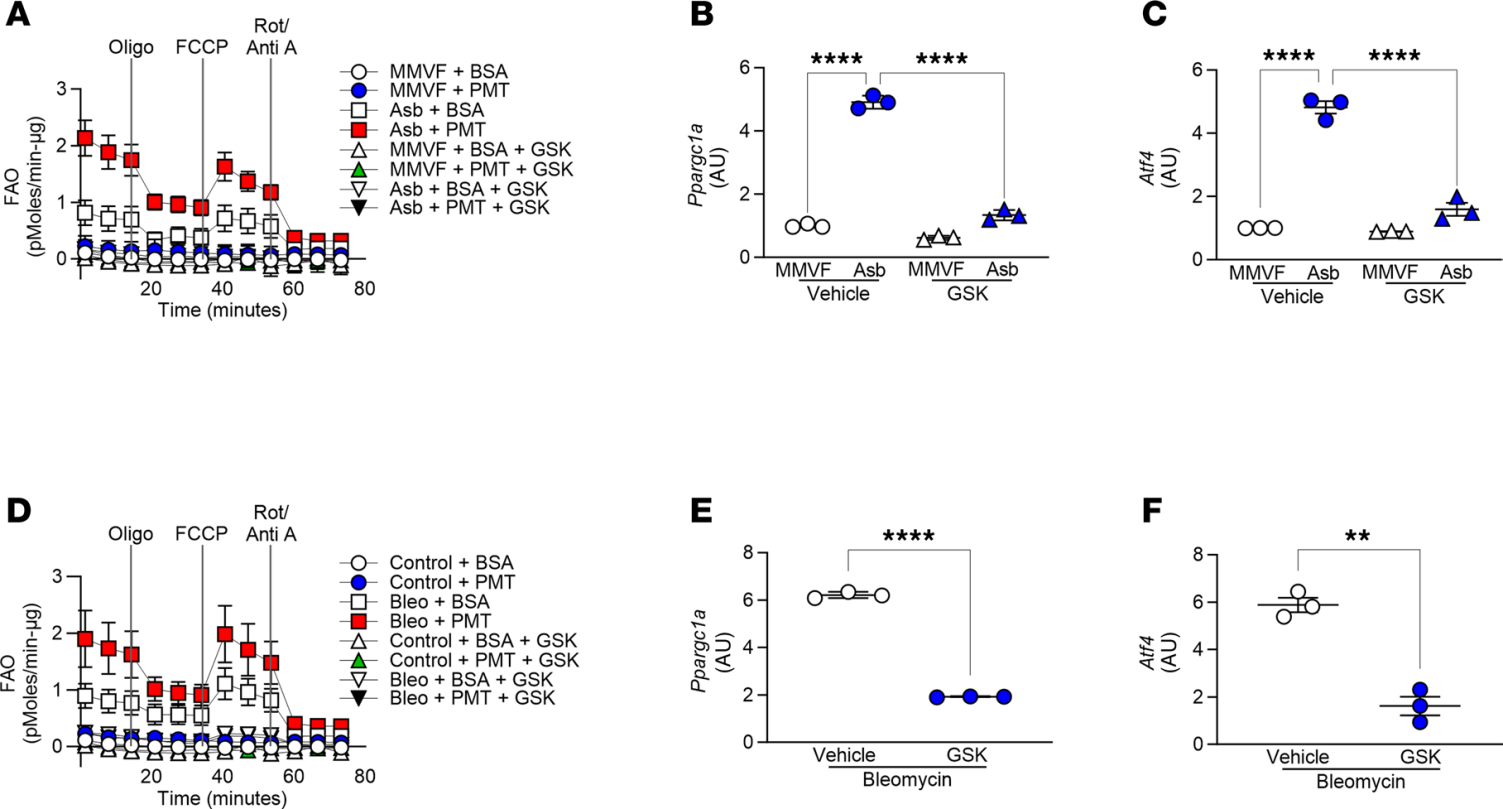
Pharmacological inhibition of PERK abrogates FAO in mice with established fibrosis. Thirteen days after exposure, GSK (30 mg/kg i.p.) was administered daily to mice. (**A**) FAO in BAL macrophages from asbestos- or MMVF-exposed mice was measured by OCR with the addition of BSA or BSA:palmitate (PMT) using Seahorse XF96 bioanalyzer (Agilent Technologies) (*n* = 4–5). min-μg, minute/μg protein. Total RNA was isolated from lung macrophages and subjected to real-time PCR for (**B**) *Ppargc1a* (*n* = 3) and (**C**) *Atf4* mRNA expression (*n* = 3). (**D**) FAO in macrophages from control or bleomycin-exposed mice was measured by OCR with the addition of BSA or PMT using Seahorse XF96 bioanalyzer (*n* = 3–5). Total RNA was isolated from lung macrophages and subjected to real-time PCR for (**E**) *Ppargc1a* (*n* = 3) and (**F**) *Atf4* mRNA expression (*n* = 3). Data shown as mean ± SEM. One-way ANOVA with Tukey’s post hoc comparison in **B** and **C**. Two-tailed Student’s *t* test in **E** and **F**. **P ≤ 0.01, ****P ≤ 0.0001. (See also Supplemental Figure 6.)

Linking PERK to metabolic reprogramming in vivo, macrophages from asbestos-injured mice had increased FAO, and the addition of palmitate further increased FAO (Figure 7A and Supplemental Figure 6B). GSK significantly reduced metabolic reprogramming to FAO in macrophages from asbestos-injured mice in the presence or absence of palmitate. In support of GSK reducing FAO, *Ppargc1a* expression was significantly reduced in GSK-treated mice exposed to asbestos (Figure 7B). Furthermore, *Atf4* was significantly reduced to control levels in macrophages from asbestos-injured mice (Figure 7C), supporting the effect of GSK on inhibiting PERK.

GSK abrogated FAO in macrophages from bleomycin-injured mice in a similar manner as seen in the asbestos-injured mice (Figure 7D and Supplemental Figure 6C). Moreover, *Ppargc1a* and *Atf4* expression was significantly decreased compared with the vehicle in bleomycin-injured mice (Figure 7, E and F).

### PERK regulates macrophage pro-fibrotic gene expression.

**Figure 8:**
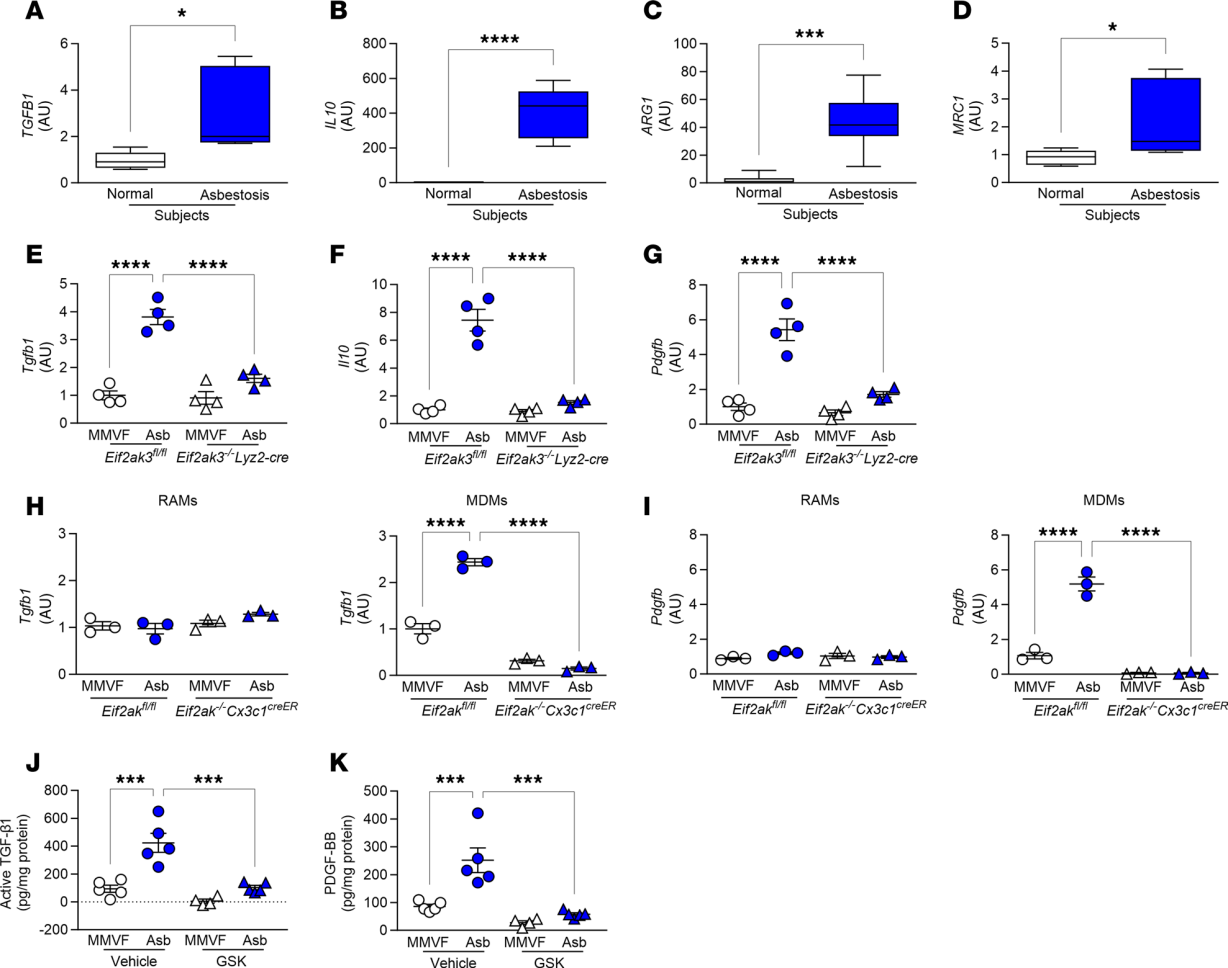
PERK regulates macrophage pro-fibrotic gene expression. Lung macrophages from normal and asbestosis humans were obtained by BAL. mRNA expression of (**A**) *TGFB1* (*n* = 5), (**B**) *IL10* (*n* = 5–7), (**C**) *ARG1* (*n* = 6), and (**D**) *MRC1* (*n* = 6) in humans. (**E**) *Tgfb1* (*n* = 4), (**F**) *Il10* (*n* = 4), and (**G**) *Pdgfb* (*n* = 4) mRNA expression in BAL isolated at day 21 from exposed *Eif2ak3^fl/fl^* and *Eif2ak3^–/–^* *Lyz2-cre* mice. (**H**) *Tgfb1* (*n* = 3) and (**I**) *Pdgfb* (*n* = 3) mRNA expression in FACS-sorted BAL cells isolated at day 21 from exposed *Eif2ak3^fl/fl^* and *Eif2ak3^–/–^* *Cx3cr1^creER^* mice. (**J**) Active TGF-β1 (*n* = 4–5) and (**K**) PDGF-BB (*n* = 4–5) in BAL fluid harvested at day 21 from WT mice administered GSK 13 days after exposure to MMVF or asbestos. Data shown as mean ± SEM. Two-tailed Student’s *t* test in **A**–**D**. One-way ANOVA with Tukey’s post hoc comparison in **E**–**K**. *P ≤ 0.05, ***P ≤ 0.001, and ****P ≤ 0.0001. (See also Supplemental Figure 7.)

PERK-mediated metabolic reprogramming in lung macrophages was associated with an increase in pro-fibrotic genes. Lung macrophages isolated from humans with asbestosis showed a significant induction in *TGFB1*, *IL10*, *ARG1*, and *MRC1* gene expression compared with healthy humans (Figure 8, A–D). Similarly, lung macrophages from asbestos-injured *Eif2ak3^fl/fl^* mice had increased *Tgfb1*, *Il10*, and *Pdgfb*, while levels in asbestos-exposed *Eif2ak3^–/–^* *Lyz-cre* mice were near those of the controls (Figure 8, E–G).

To investigate the macrophage subset responsible for the increase in pro-fibrotic genes, we FACS-sorted RAMs and MDMs from exposed *Eif2ak3^fl/fl^* and *Eif2ak3^–/–^* *Cx3cr1^creER^* mice. MDMs from asbestos-injured *Eif2ak3^fl/fl^* mice showed significantly increased *Tgfb1* and *Pdgfb* compared with MMVF-exposed *Eif2ak3^fl/fl^* mice (Figure 8, H and I). The absence of PERK in MDMs from *Eif2ak3^–/–^* *Cx3cr1^creER^* mice mediated a significant reduction in expression of *Tgfb1* and *Pdgfb* compared with MMVF-exposed *Eif2ak3^–/–^* *Cx3cr1^creER^* mice.

Administering the PERK inhibitor, GSK, after fibrosis led to a reduction in active TGF-β1 and PDGF-BB in the BAL fluid from asbestos-exposed mice (Figure 8, J and K). Similar results were obtained in vitro. GSK treatment inhibited an increase in pro-fibrotic gene expression in macrophages after asbestos exposure (Supplemental Figure 7, A–E). Taken together, our observations demonstrate that PERK was activated in lung MDMs during fibrosis, suggesting it is a druggable target to reverse fibrotic remodeling. Moreover, GSK reversed established fibrosis, at least in part, by abrogating PERK-mediated metabolic reprogramming to FAO and pro-fibrotic polarization in MDMs.

## Discussion

ER stress has a critical role in pathogenesis of fibrotic diseases, including liver fibrosis (36), kidney fibrosis (37), and lung fibrosis (28). In the lung, ER stress in AECs (38) and fibroblasts (28) has been well described to have a role in fibrotic remodeling. In lung macrophages, ER stress and activation of UPR are associated with pro-fibrotic polarization (31). PERK has a central role in the immunosuppressive function of macrophages (34); however, IRE1α suppresses the alternative activation of macrophages (31). Another study showed that mice with a global knockout of CHOP were protected from bleomycin-induced fibrosis, in part, by reducing pro-fibrotic polarization of macrophages (39). In a repetitive bleomycin-induced mouse model of pulmonary fibrosis, CHOP expression was localized to type II AECs within fibrotic regions and was implicated in driving AEC apoptosis (40). Similarly, in idiopathic pulmonary fibrosis individuals, CHOP expression was detected in hyperplastic type II AECs, where it colocalized with markers associated with enhanced glycolysis. The role of ER stress, specifically in lung macrophages and in metabolic reprogramming, in asbestos-induced fibrosis has not been described.

Macrophages are critical for lung fibrosis development by initiation of an immune response and their polarization into phenotypically distinct subpopulations. Our group and others have demonstrated a predominant number of recruited MDMs in fibrotic lungs that are the macrophage subset responsible for driving fibrosis development and progression (13, 24, 33). ER stress and UPR have been linked to pro-fibrotic activation of macrophages (30, 31). Targeting pro-fibrotic macrophages has provided strong evidence in several preclinical studies that a phenotypic change from a pro-fibrotic macrophage to an antifibrotic phenotype could reverse established fibrosis (9, 16, 33). Our data support this notion because there were a reduction of MDMs and decreased production of pro-fibrotic factors in *Eif2ak3^–/–^* *Lyz2-cre* mice, which were protected from pulmonary fibrosis.

One mechanism by which ER stress induces glycolysis in brown adipocytes in response to cold stress is through ATF4-mediated reduction of PGC-1α levels (41). This is driven by the competitive binding of ATF4 with CREB at a CRE site on the *Ppargc1a* promoter. ATF4 also inhibited *Ppargc1a* transcription during ER stress in skeletal muscle (42). One study showed that PGC-1α interacted with and suppressed XBP1 function, thereby enhancing gluconeogenesis (43). Moreover, IRE1α has been shown to degrade *Ppargc1a* in adipocytes via its RNase function (44).

Metabolic reprogramming has a crucial role in disease progression in many conditions, such as cancer, obesity, congestive heart failure, liver fibrosis, and chronic lung diseases. A key feature of macrophage activation during repair involves metabolic reprogramming to OXPHOS and FAO (45, 46). The metabolic reprogramming in macrophages is necessary to support long-term cellular activities in lung remodeling, as well as increase the apoptotic resistance that is seen in these cells (47, 48). ATF4 is known to regulate carbohydrate metabolism (49), while ATF4-knockout mice had increased OXPHOS and decreased ATP production because of expression of UCP1 (50). PGC-1α increases the enzymatic capacity for FAO, mitochondrial biogenesis, and oxidative metabolism (51). Our data showed that the molecular mechanism of the PERK/ATF4 pathway in MDMs is distinct in that it mediated a positive effect on PGC-1α expression whereby ATF4 increased *Ppargc1a* transcription.

Studies have identified involvement of PERK in mitochondrial dynamics to meet metabolic needs (35). Specifically, loss of PERK reduced mitochondrial respiration, resulting in a significant reduction of OCR in bone marrow–derived macrophages with an M2 phenotype (34); however, the mechanism of this reduction was not determined. The PERK/ATF4 pathway drives amino acid biosynthesis in autosomal dominant polycystic kidney disease (52), and it augments antitumor activity of T cells by increasing mitophagy (53).

PERK and eIF2α activation in ER stress promotes a pro-survival pathway in chronic myeloid leukemia (54), and there is evidence that ER stress induces apoptosis resistance through activation of PERK (55). We previously showed that macrophages, especially MDMs, are resistant to apoptosis (15–18). MDMs displayed apoptosis resistance in response to asbestos-induced ER stress and during fibrotic progression. These observations suggest that PERK mediates apoptosis resistance and progression of fibrotic remodeling via metabolic reprogramming to FAO.

The limitations of the study include determining the mechanism by which IRE1α is not activated after exposure to asbestos. Studies indicate PERK inhibits IRE1α by mediating the dephosphorylation and inactivation of IRE1α (56). While our data strongly indicate that *ATF4* regulates *Ppargc1a* expression, other transcription factors that are modulated by ER stress may also play a role. Although PERK and asbestos exposure altered *ATF3* expression, *ATF3* did not regulate expression of *Ppargc1a*. PERK clearly has a critical effect in lung macrophages during fibrosis, but the role of PERK or its inhibition in other cell types involved in fibrosis may have a prominent contribution in established fibrosis. Moreover, the absence of PERK in *Eif2ak3^–/–^* *Cx3cr1^creER^* mice led to a reduction in the number of MDMs present in the lung. The human data support the in vitro and in vivo studies, but the complete translation into humans with established fibrosis will require more in-depth investigations in ER stress. These limitations underscore areas of future research.

PERK inhibitors have been utilized as therapeutics in different disease models (34, 57, 58), and GSK is a highly selective inhibitor of PERK and had some antiinflammatory effects. With regard to humans, our study excluded individuals taking prednisone or other antiinflammatory medications, except aspirin. PERK was not inhibited in individuals taking aspirin. In *Mycobacterium tuberculosis* infection, GSK reduced PERK phosphorylation, as well as decreased fibrous tissue hyperplasia, inflammatory infiltration, and the bacterial load in the lung tissue (57). A PERK inhibitor ameliorated bleomycin-induced lung fibrosis through STING/PERK/eIF2α signaling with restored lung architecture and reduced collagen expression (58); however, treatment was started during the inflammatory stage, 7 days after bleomycin exposure. It is unclear if PERK inhibition decreased inflammation to reduce lung injury, as fibrosis is not evident until day 10 after bleomycin- or asbestos-induced injury (7, 14). Our data showed that PERK inhibition reversed established fibrosis in an asbestos- and bleomycin-induced injury model by reduced expression of ATF4 and attenuation of FAO in lung macrophages. These observations strongly suggest that PERK is a potential therapeutic target in pulmonary fibrosis.

## Methods

Further information can be found in Supplemental Methods.

### Sex as a biological variable.

Our study examined male and female participants as well as male and female animals. Similar data were obtained in both sexes and so were not reported separately.

### Humans.

Human BAL cells were obtained as previously described (6, 15, 17, 59, 60) from healthy and asbestosis humans under an approved protocol by the Human Subjects Institutional Review Board of University of Alabama at Birmingham (3000012729 and 300004607). All studies followed the Declaration of Helsinki principles, and human BAL samples were used for research only. All participants provided prior written informed consent to participate in the study. Healthy volunteers had to meet the following criteria: (i) age between 35 and 85 years, (ii) no history of cardiopulmonary disease or other chronic disease, (iii) no prescription or nonprescription medication except oral contraceptives and aspirin, (iv) no recent or current evidence of infection, and (v) lifetime nonsmoker. Asbestosis participants had to meet the following criteria: (i) forced vital capacity at least 50% predicted; (ii) current nonsmoker; (iii) no recent or current evidence of infection; (iv) no treatment with prednisone or other antiinflammatory medications except aspirin; (v) evidence of restrictive physiology on pulmonary function tests; (vi) usual interstitial pneumonia on high-resolution chest computed tomography; (vii) occupational or environmental exposure to asbestos and detection of asbestos bodies in BAL; and (viii) no evidence of cardiac conducting abnormalities, severe cardiovascular disease, history of malignancy, or renal disease. Fiber-optic bronchoscopy with BAL was performed after participants received local anesthesia. Three subsegments of the lung were lavaged with five 20 mL aliquots of normal saline, and the first aliquot in each was discarded. The percentage of macrophages was determined by Wright-Giemsa stain and varied from 90% to 98%.

### Mice.

The University of Alabama at Birmingham Institutional Animal Care and Use Committee approved the animal experiments (nos. 23028, 22760, and 21969). All the experiments were performed as per the NIH *Guide for the Care and Use of Laboratory Animals* (National Academies Press, 2011). C57BL/6 WT or *Eif2ak3^fl/fl^*, *Eif2ak3^−/−^* *Lyz2-cre*, and *Eif2ak3^−/−^* *Cx3cr1^creER^* mice were used. The *Eif2ak3^fl/fl^* and *Eif2ak3^–/–^* *Lyz2-cre* mice were a gift from Stanley Huang (Case Western Reserve University, Cleveland, Ohio, USA; now at The Ohio State University, Columbus, Ohio, USA). *Eif2ak3^−/−^* *Cx3cr1* mice were generated by breeding *Eif2ak3^fl/fl^* and *Cx3cr1^creER^* mice (Jackson Laboratory, 020940).

### Cell culture.

All cell lines were purchased from ATCC and cultured as recommended.

### FACS.

Hierarchical gating strategy was used to represent the RAMs as CD45^+^CD11b^+/–^Ly6G^–^CD64^+^Ly6c^–^Siglec-F^hi^ and MDMs as CD45^+^CD11b^+/–^Ly6G^–^CD64^+^Ly6c^–^Siglec-F^lo^. Data were acquired on LSR II (BD Biosciences) using BD Biosciences FACSDiva software (version 8.0.1). Data were analyzed using FlowJo (FlowJo LLC) software (Version 10.5.0).

### Plasmids, transfections, siRNA, and luciferase assays.

The pcDNA3.1 (Invitrogen), PERK^WT^ (plasmid 21814), PERK^DN^ (plasmid 36954), and IRE1α^WT^ (plasmid 20744) vectors were purchased from Addgene. *Ppargc1a* gene expression was evaluated using a luciferase reporter plasmid from Bruce Spiegelman purchased from Addgene (plasmid 8887) (61). Plasmids were transfected with X-tremeGene 9 Transfection Reagent (06365809001; Roche), according to the manufacturer’s protocol. After 24–72 hours, cells were exposed to vehicle or asbestos. All siRNAs were purchased from Integrated DNA Technologies. Cells were transfected using Dharma-FECT 4 (T-2004; Dharmacon) or Dharma-FECT 2 (T-2002; Dharmacon), according to the manufacturer’s protocol. *Ppargc1a* promoter reporter vector was used to generate mutants to prevent binding of *Atf4*. *Atf4* binding site -1357 GTGACGTCA -1347 was mutated to -1357 ATAATGCTA -1347 using a site-directed mutagenesis kit from Agilent Technologies using the primers forward 5′-GCTGCCTCGGAATAATGCTAGGAGTTTGTGCAG-3′ and reverse 5′-CTGCACAAACTCCTAGCATTATTCCGAGGCAGC-3′. The DNA sequence was confirmed by Heflin Center Genomics Core at University of Alabama at Birmingham.

### Immunoblot analysis.

Immunoblot analysis was performed as previously described (6). Densitometry was performed using ImageJ software (NIH).

### Confocal imaging.

The fluorescence staining protocol was described previously (15, 33). Briefly, BAL cells were fixed with 4% paraformaldehyde at room temperature for 45 minutes followed by permeabilization for 3–5 minutes in ice-cold buffer (0.1% sodium citrate and 0.1% Triton X-100 in distilled water). Cells were blocked at room temperature and stained. The Nikon A1 confocal microscope was used for imaging, and all the images were quantitated using ImageJ.

### IHC.

IHC protocol was previously described (33, 62). Briefly, lung tissue sections from mice (4 μm thick) were prepared and fixed in 10% formalin followed by paraffin-embedded tissue sectioning. Tissue sections were deparaffinized by incubating at 60°C for 30 minutes and washed in xylene for 5 minutes twice. Tissues were rehydrated with gradient series of ethanol (absolute; 95%, 90%, 80%, and 70% in water) with 3 minutes for each incubation followed by blocking in PBS containing 10% BSA and 10% normal goat serum; then tissue sections were stained. The Nikon A1 confocal microscope was used for imaging, and all images were quantitated using ImageJ.

### Real-time quantitative PCR.

Total RNA was isolated using TRIzol reagent (15596018; Thermo Fisher Scientific) and reverse-transcribed with iScript reverse transcription kit (170-8891; Bio-Rad). Expression of mRNA was determined by real-time quantitative PCR using iQ SYBR Green supermix (170-8882; Bio-Rad). Data were calculated by using the ^ΔΔ^Ct method. Measurements were normalized to HPRT (human) or β-actin (mouse) and expressed in arbitrary units.

### ChIP assay.

The ChIP assay was performed using the SimpleChIP enzymatic chromatin IP kit (9002S; Cell Signaling Technology) according to the manufacturer’s instructions and as previously described (59, 63). Briefly cross-linked chromatin preparations were used for input controls (2% of total) or for immunoprecipitation of ATF3 (D2Y5W) anti-rabbit monoclonal (33593; Cell Signaling Technology), ATF4 anti-rabbit polyclonal (10835-1-AP; Proteintech), histone H3 (D1H2) XP Rabbit (4499; Cell Signaling Technology) as a positive control, or normal rabbit IgG (2729; Cell Signaling Technology) as a negative control. Protein-DNA complexes were eluted and the chromatin was subjected to reversal of cross-links followed by DNA purification (14209S; Cell Signaling Technology). Real-time PCR was performed using purified DNA.

### Statistics.

Statistical comparisons were performed using 1-way ANOVA with a Tukey’s post hoc test or 2-tailed Student’s *t* test. All statistical analysis was expressed as ±SEM with *P* < 0.05 being considered significant. GraphPad Prism statistical software was used for all analysis.

### Study approval.

We obtained BAL cells from normal and asbestosis humans under a protocol (3000012729 and 300004607) approved by the Human Subjects Institutional Review Board of the University of Alabama at Birmingham. Human BAL specimens were used for research only. All participants provided prior written consent to participate in the study. Animal experiments were approved by the University of Alabama at Birmingham Institutional Animal Care and Use Committee under protocols 23028, 22760, and 21969 and were performed in accordance with the NIH *Guide for the Care and Use of Laboratory Animals* (National Academies Press, 2011).

### Data availability.

Supporting data for all data points presented in the graphs are provided in the Supporting Data Values file.

## Author contributions

JP performed experiments, analyzed data, and wrote the first draft of the manuscript; JLLC performed experiments, analyzed data, wrote and edited the manuscript, and assisted in figure preparation; MHP generated mutant *Ppargc1a* luciferase; CH provided some of the human samples; NP managed and genotyped the mouse colony; and ABC provided reagents, generated the overall hypothesis, provided direction for the study, analyzed data, and edited the manuscript in its final version.

## Supplementary Material

Supplemental data

Unedited blot and gel images

Supporting data values

## Version 1 04/10/2025

In-Press Preview

## Version 2 05/22/2025

Electronic publication
