# Supplementary material for: The PERK/ATF4 pathway is required for metabolic reprogramming and progressive lung fibrosis
Source: JCI Insight. 2025 Apr 10;10(10):e189330. doi: 10.1172/jci.insight.189330 (PMC12128959; doi:10.1172/jci.insight.189330)
Supplement: Supplemental data [file jciinsight-10-189330-s019.pdf]

Supplemental Information

The PERK-ATF4 pathway is required for metabolic reprogramming and progressive lung fibrosis

Jyotsana Pandey, Jennifer L. Larson-Casey, Mallikarjun H. Patil, Chao He, Nisarat Pinthong, and A. Brent Carter

Supplemental Methods

Mice. Mice were administered corn oil dissolved tamoxifen for 5 days (20mg/kg, i.p.). Mice where further maintained, genotyped, and propagated at animal facilities at UAB as per IACUC guidelines. Six-twelve-week-old male and female mice were intratracheally administered with 100 µg of chrysotile asbestos (gift from Dr. Peter Thorne, University of Iowa) or man-made vitreous fiber (MMVF), as control, suspended in 50 µL 0.9% saline solution, or saline or bleomycin (1.5 U/kg). After being anesthetized with 3% isoflurane using a precision Fortec vaporizer (Cyprane). On day 15-21 after exposure, mice were administered vehicle or GSK2656157 (S7033; Selleckchem; 30mg/kg, i.p.) daily for 7 consecutive days. Clodronate-loaded liposomes or control liposomes (50 µl, Liposoma Technology) were administered i.t. seven days after asbestos exposure. BAL was performed and lungs were extracted 21 days after exposure, unless stated otherwise. The presence of asbestos fibers was not assessed in mouse lungs.

Cell culture. All cell lines were purchased from ATCC and cultured as recommended. Mouse alveolar macrophage (MH-S, CRL-2019) cells are derived by SV40 transformation of an adherent cell enriched population of mouse alveolar macrophages from BALB/c mice. Human monocyte (THP-1, TIB-202) cells were isolated from peripheral blood from an acute monocytic leukemia patient. Cells were maintained in culture at 37˚C and 5% CO_2 and grown in RPMI-1640 medium (Thermo Fisher Scientific) with the following supplements: 10% fetal bovine serum, and penicillin/streptomycin. Mouse lung epithelial cells (MLE 12, CRL-2110) were cultured in HITES medium supplemented with 2% fetal

bovine serum. Human lung fibroblast cells (IMR-90, CCL-186) were cultured in EMEM media supplemented with 10% FBS. All experiments were performed with 0.5% serum supplement. Chrysotile asbestos (10 µg/cm^2) was used to expose cells, as previously described (1). Cells were treated with GSK2656157 (5 µM, unless stated otherwise) for 6 h prior to asbestos exposure.

Fluorescence-activated cell sorting (FACS). BAL cells were blocked with 1% BSA containing TruStain fcX (anti-mouse CD16/32) antibody (101319; BioLegend), followed by staining with antibodies. Antibodies used: Rat anti-mouse CD45-PE (12-0451-82; eBiosciences), LIVE Dead-eflour506 (65-0866; Invitrogen), Rat anti-mouse CD11b-APC-Cy7 (101225; BioLegend), anti-mouse CD64-PE-Cy7 (139313; BioLegend), Rat anti-mouse Ly6G-AF700 (561236; BD), Rat anti-mouse Siglec F-APC (155507; BioLegend), Rat anti-mouse Ly6C: eflour450 (48-5932-82; Invitrogen), Annexin V-FITC (640906; BioLegend), and PI (V13242; Molecular Probes). Hierarchical gating strategy was used to represent the resident alveolar macrophages as CD45$^+$CD11b$^{+/-}$Ly6G$^-$CD64$^+$Ly6c$^-$ Siglec F^{hi} and monocyte-derived macrophages as CD45$^+$CD11b$^{+/-}$Ly6G$^-$CD64$^+$Ly6c$^-$ Siglec F^{low}. Data was acquired on LSR II (BD Biosciences) using BD FACS DIVA software (version 8.0.1). Data was analyzed using FlowJo (FlowJo LLC) software (Version 10.5.0).

Plasmids, transfections, small interfering RNA (siRNA), luciferase assays. The pcDNA3.1 (Invitrogen), PERK^{WT} (21814), PERK^{DN} (36954), and IRE1α^{WT} (20744) vectors and *Ppargc1a* luciferase reporter plasmid (8887) were purchased from Addgene. Plasmids were transfected with X-tremeGene 9 Transfection Reagent (06365809001;

Roche), according to the manufacturer's protocol. After 24–72 h, cells were expose to vehicle or asbestos. All siRNA were purchased from Integrated DNA Technologies with the following sequences: mouse *Eif2ak3* 5'-GAUUGGAAGGUC AUGGCGUUUAGTA-3' and 5'-UACUAAACGCCAUGACCUUCCAAUCAG-3', human *ATF4* 5'-AAAUUCUCAGAACAGCUAACCUCTA and UAGAGGUUAGCUGUUCUGAGAAUUUCA-3'. Cells were transfected using Dharma-FECT 4 (T-2004; Dharmacon) or DharmaFECT 2 (T-2002; Dharmacon), according to the manufacturer's protocol. Eight hours following transfection, media was replaced, and cells were allowed to recover for 24–72 h. Samples were collected post asbestos exposure. Renilla and firefly luciferase activity was determined in cell lysates using the Dual Luciferase reporter assay kit (Promega) as per manufacturer's protocol and normalized to control (firefly). *Ppargc1a* promoter reporter vector was used to generate mutants to prevent binding of *Atf4*. *Atf4* binding site -1357 GTGACGTCA -1347 was mutated to -1357 ATAATGCTA -1347 using site directed mutagenesis kit from Agilent Technologies using the primers: forward 5'-GCTGCCTCGGAATAATGCTAGGAGTTTGTGCAG-3' and reverse 5'-CTGCACAAACTCCTAGCATTATTCCGAGGCAGC-3'. The DNA sequence was confirmed by Heflin Center Genomics Core at UAB.

Isolation of nuclei. Nuclei were isolated as previously described (2, 3) from cell lines and BAL cells.

Immunoblot analysis. Primary antibodies used were as follows: PGC-1α anti-rabbit monoclonal (2178S), Lamin A/C anti-rabbit polyclonal (2032), PERK anti-rabbit

monoclonal (3192S), phospho-eIF2alpha anti-rabbit monoclonal (9721S), eIF2α (D7D3) anti-rabbit monoclonal (5324), phospho-PERK (16F8) anti-rabbit monoclonal (3179S), IRE1alpha anti-rabbit monoclonal (3294S), ATF4 (D4B8) anti-rabbit monoclonal (11815), ATF3 (D2Y5W) anti-rabbit monoclonal (33593), and CHOP anti-mouse monoclonal (2895S) were from Cell Signaling Technology; ATF4 polyclonal (10835-1-AP), PERK/EIF2AK3 polyclonal (20582-1-AP), and IRE1; ERN1 polyclonal (27528-1-AP) were from Proteintech; phospho-PERK anti-rabbit polyclonal (PA5-40294) and phospho-IRE1 alpha anti-rabbit polyclonal (PA1-16927) were from Invitrogen; and β-actin anti-mouse monoclonal (A5441) was from Millipore. Densitometry was performed using ImageJ software.

Confocal imaging. Fluorescence staining protocol was described previously (4, 5). Briefly, BAL cells were fixed with 4% paraformaldehyde at room temperature for 45 min followed by permeabilization for 3-5 min in ice-cold buffer (0.1% sodium citrate and 0.1% Triton X-100 in distilled water). Cells were blocked at room temperature for 1 h in DPBS with 10% BSA and 10% Goat serum and then incubated with TruStain FcX™ anti-mouse (156604; BioLegend) or TruStain FcX™ anti-human CD16/32/64 (422302; BioLegend) to block non-specific binding of immunoglobulin to the Fc receptors. Further incubations were performed with phospho-PERK (16F8) anti-rabbit monoclonal (3179S; Cell Signaling), F4/80-PE anti-mouse monoclonal (123109; BioLegend), phospho-eIF2α anti-rabbit polyclonal (9721S; Cell Signaling), goat anti-rabbit IgG-FITC (4030-02; Southern Biotech), goat anti-mouse IgG-TRITC (1030-03; Southern Biotech) for 1 hr with 2% BSA and 2% goat serum BSA in each antibody for staining. DAPI (157577; MP Biologicals)

was used for nuclear staining for 10 mins. Cells were fixed with cover slide using ProLong™ Gold Antifade Mountant (P36930; Thermo Fisher Scientific) followed by confocal microscopy. The Nikon A1 confocal microscope was used for imaging and all the images were quantitated using ImageJ (NIH).

Immunohistochemistry (IHC). IHC protocol was previously described (4, 6). Briefly, lung tissue sections from mouse (4-μm-thick) were prepared and fixed in 10% formalin followed by paraffin embedded tissue sectioning. Tissue sections were deparaffinized by incubating at 60 °C for 30 min and washed in xylene for 5 min twice. Tissues were rehydrated with gradient series of ethanol (absolute; 95%, 90%, 80%, and 70% in water) with 3 min each incubation followed by blocking in PBS containing 10% BSA and 10% normal goat serum then incubated with TruStain FcX™ PLUS anti-mouse CD16/32 (156604; BioLegend) to block non-specific binding of immunoglobulin to the Fc receptors. Tissue sections were stained with phospho-PERK (16F8) anti-rabbit monoclonal (3179S; Cell Signaling), phospho-PERK (Thr981) (4A3A10) anti-mouse monoclonal (MA5-50284; Invitrogen), F4/80-PE anti-mouse monoclonal (123109; BioLegend), prosurfactant protein C (4A10) anti-mouse monoclonal (H00006440-M01; Novus Biologicals), SMA anti-mouse monoclonal (03-61001; American Research Products), collagen type I anti-rabbit polyclonal (AB765P; Millipore), goat anti-rabbit IgG-FITC (4030-02; Southern Biotech), goat anti-rabbit IgG-TRITC (4050-03; Southern Biotech), goat anti-mouse IgG-FITC (1036-02; Southern Biotech), goat anti-mouse IgG-TRITC (1030-03; Southern Biotech), TruStain FcX™ PLUS anti-mouse CD16/32 (156604; BioLegend). Antibodies were diluted in 2% BSA and 2% normal goat serum for 1h each and then counterstained with

DAPI (157577; MP Biologicals). Tissue sections were fixed with cover slide using ProLong™ Gold Antifade Mountant (P36930; Thermo Fisher Scientific) followed by confocal microscopy. The Nikon A1 confocal microscope was used for imaging and all images were quantitated using ImageJ (NIH).

Oxygen consumption rate (OCR). OCR measurement by a Seahorse XF96 bioanalyzer (Seahorse Bioscience) was performed as described (5).

Caspase-3 activity.

Caspase-3 activity was quantitated with the EnzCheck Caspase-3 Assay Kit #2 (MP13184; Molucular Probes) according to the manufacturer's instructions and as previously described (5). Ac-DEVD-CHO, an inhibitor of caspase-3, was used.

Real-time quantitative PCR. Total RNA was isolated using Trizol reagent (15596018; Thermo Fisher Scientific) and reverse transcribed with iScript reverse transcription kit (170-8891; Bio-Rad). Expression of mRNA was determined by real-time quantitative PCR using iQ SYBR green supermix (170-8882; Bio-Rad). Data were calculated by using the $^{\Delta\Delta}$Ct method. Measurements were normalized to HPRT (human) or β-actin (mouse) and expressed in arbitrary units. Primers used were purchased from Integrated DNA Technology:

Gene	Forward Sequence	Reverse Sequence
ARG1	TTC TCA AAG GGA CAG CCA CG	TAG GGA TGT CAG CAA AGG GC
ATF3	GCA CCT TGC CCC AAA ATC A	TCC TTG ACA AAG GGC GTC AG
ATF4	GCC ATT TCT ACT TTG CCC GC	GGC GCT CGT TAA ATC GCT TC
CPT1A	CAT CAT CAC TGG CGT GTA CC	TTG GCG TAC ATC GTT GTC AT
HPRT	AGC CCT GGC GTC GTG ATT AGT GA	TGT CCC CTG TTG ACT GGT CAT TAC A
IL10	GAT CCA GTT TTA CCT GGA GGA G	CCT GAG GGT CTT CAG GTT CTC

MRC1	CC TCC TTG TCC TGG TCT G	GCT TCA GGTT CGC TGA TGT ATT
PPARGC1A	GAG TGT GTG CTC TGT GTC ACT	CAG CAC ACT CGA TGT CAC TCC AT
TGFB1	CGT GGA GCT GTA CCA GAA ATC	CAC AAC TCC GGT GAC ATC AA
Arg1	CAG AAG AAT GGA AGA GCT AG	CAG ATA TGC AGG GAG TCA CC
Atf4	TGG GGC CTT TAG GAC GAT CT	ACT GCT GCT GGA TTT CGT GA
β-actin	GCC TTC CTT CTT GGG TAT GG	CAG CTC AGT AAC AGT CCG CC
Chil3	TGT TCT GGT GAA GGA AAT GCG	CGT CAA TGA TTC CTG CTC CTG
Ccl2	CAG GTC CCT GTC ATG CTT CT	GTG GGG CGT TAA CTG CAT CT
Cpt1a	GCT GGG CTA CTC AGA GGA TG	ACT GTA GCC TGG TGG GTT TG
Eif2ak3	GGT GGA GTC CC TGC TCG AAT	GCG TAC GCT GCA GAA GCA AG
Il6	CCC CAA TTT CCA ATG CTC TCC	CGC ACT AGG TTT GCC GAG TA
Il10	CCA GTT TTA CCT GGT AGA AGT GAT G	TGT CTA GGT CCT GGA GTC CAG CAG AC
Nos2	GTT TCT GGC AGC AGC GGC TC	GCT CCT CGC TCA AGT TCA GC
Pdgfb	CTG CCC CTC AAA AGC CTA GT	TGA CCA CCA TCC CTG TGA AAT
Ppargc1a	GGC AGT AGA TCC TCT TCA AGA TC	TCA CAC GGC GCT CTT CAA TTG
Tgfb1	CGG AGA GCC CTG GAT ACC A	TGC CGC ACA CAG CAG TTC
Tnf	CAC TTG GTG GTT TGC TAC GA	CCA CAT CTC CCT CCA GAA AA-3

Chromatin immunoprecipitation assay. The chromatin immunoprecipitation assay performed using the SimpleChIP enzymatic chromatin IP kit (9002S; Cell Signaling) according to the manufacturer’s instructions and previously described (1, 7). Briefly 4×10^6 million cells were fixed using 1% formaldehyde for 15 minutes at room temperature, followed by termination of fixation using excess glycine. Cells were spin down and washed with PBS followed by nuclei isolation. Nuclear pellets were treated with micrococcal nuclease to digest DNA to fragments. The resulting cross-linked chromatin preparations were used for input controls (2% of total) or for immunoprecipitation using 1.5μg of ATF3 (Cell Signaling) and ATF4 (Proteintech) antibodies, 10 μl histone H3 (D2B12) XP rabbit monoclonal antibody as a positive control, or 1.5 μg normal rabbit IgG antibody as a negative control. As per the protocol protein–DNA complexes were eluted, and the chromatin was subjected to reversal of cross-links followed by DNA purification (14209S; Cell Signaling). Real-time PCR was performed using purified DNA and the following primers: 5'-AAACAGGGAGCTTTGCCACT-3' and 5'-

AAAGTAGGCTGGGCTGTCAC-3' for ATF3 or ATF4 binding site on *Ppargc1a* promoter region. RPL30 primers were used for detection of the ribosomal protein gene locus.

Supplemental Figures and Legends

Supplemental Figure 1

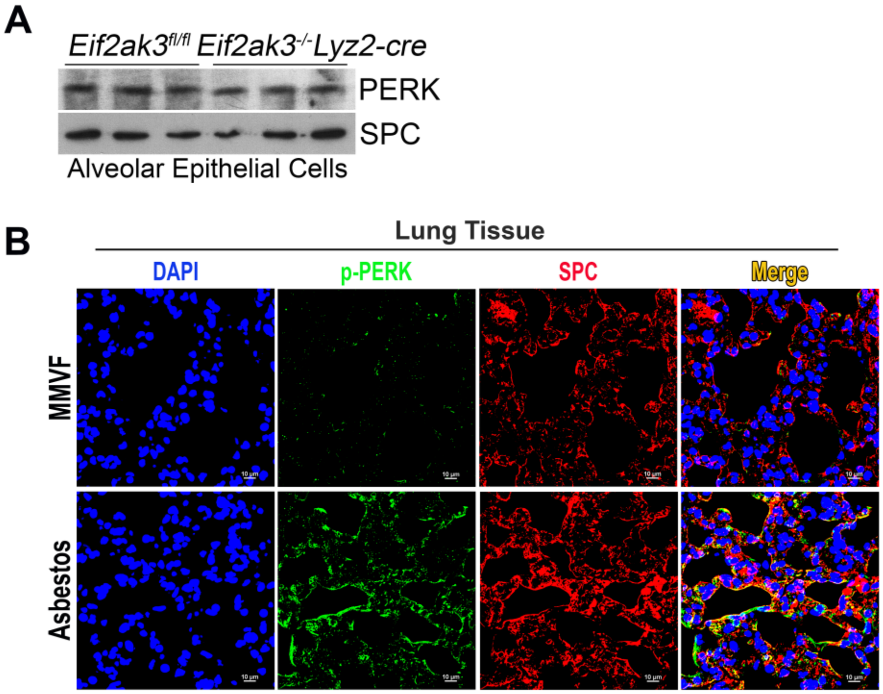

Supplemental Figure 1. PERK expression in AECs and fibroblasts. A Lungs were harvested from *Eif2ak3*$^{fl/fl}$ and *Eif2ak3*$^{-/-}$*Lyz2-cre* mice. AECs were isolated and subjected to immunoblot analysis for PERK and SPC (*n*=3). Lung tissue sections from MMVF- or asbestos-exposed mice were stained with **B** p-PERK and SPC and imaged by confocal microscopy, scale bars at 10 μm.

Supplemental Figure 2

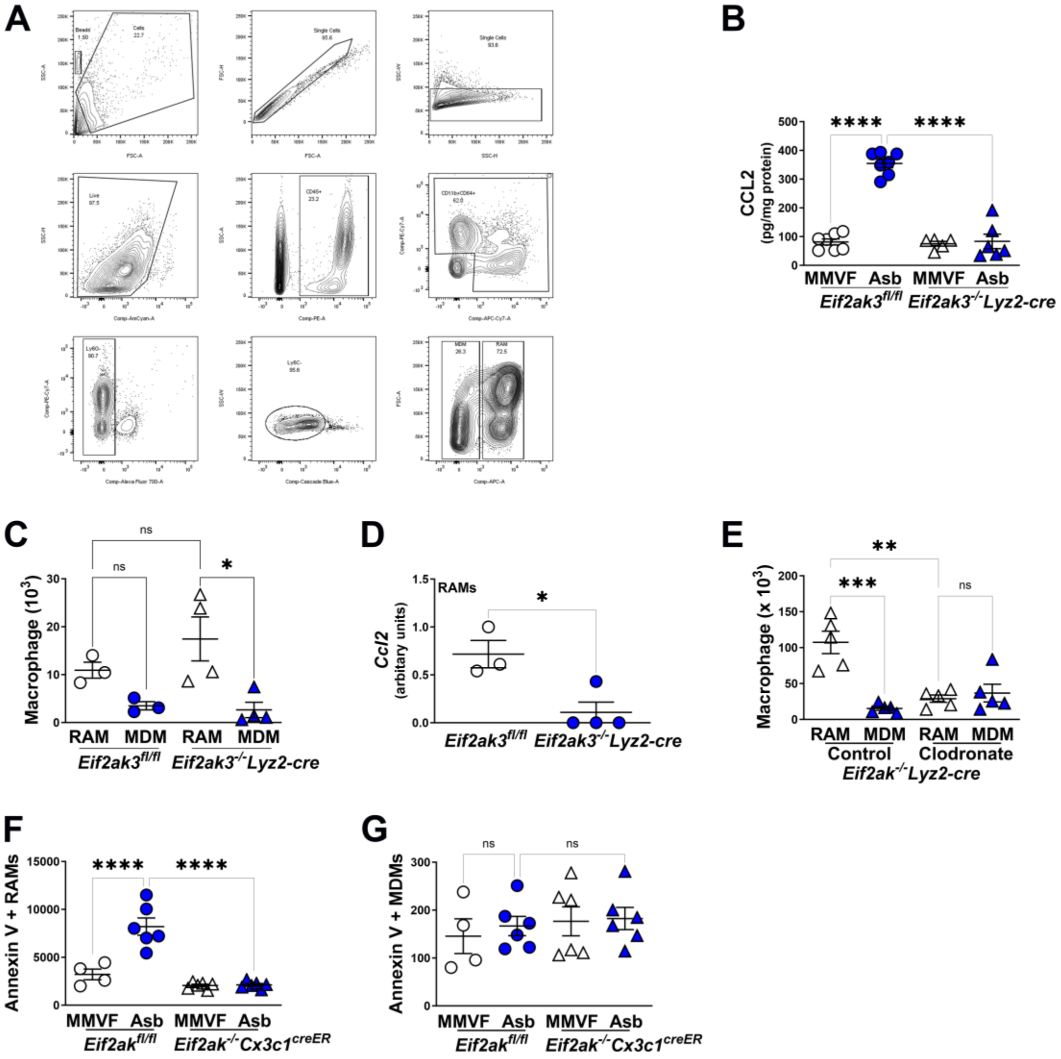

Supplemental Figure 2. PERK mediated monocyte-derived macrophage (MDMs) recruitment in asbestos-injured mice. A. Representative flow gating strategy of monocyte-derived macrophages (MDMs) and resident alveolar macrophages (RAMs) in *Eif2ak3*$^{fl/fl}$ mice exposed to asbestos. **B** CCL2 levels in BAL fluid harvested on day 21 from *Eif2ak3*$^{fl/fl}$ *and Eif2ak3*$^{-/-}$*Lyz2-cre* mice exposed to MMVF or asbestos (*n*=5-7). BAL was performed on *Eif2ak3*$^{fl/fl}$ *and Eif2ak3*$^{-/-}$*Lyz2-cre* mice 7 days after exposure to MMVF or asbestos. **C** Number of RAMs and MDMs (*n*=3-4) and **D** *Ccl2* expression in FACS-sorted RAMs from the BAL (*n*=3-4). **E** 7 days after exposure to asbestos, *Eif2ak3*$^{-/-}$*Lyz2-cre* mice were administered control or clodronate loaded liposomes (50 µl, i.t.). BAL was performed on day 21 to determine the number of RAMs and MDMs by FACS (*n*=5). Number of Annexin V positive **F** RAMs and **G** MDMs (*n*=4-6). Data shown as mean ± SEM. One-way ANOVA with Tukey's post hoc comparison in B, D-G. Two-tailed student's *t*-test in C. *$p \leq 0.05$, **$p \leq 0.01$, ***$p \leq 0.001$, and ****$p \leq 0.0001$.

Supplemental Figure 3

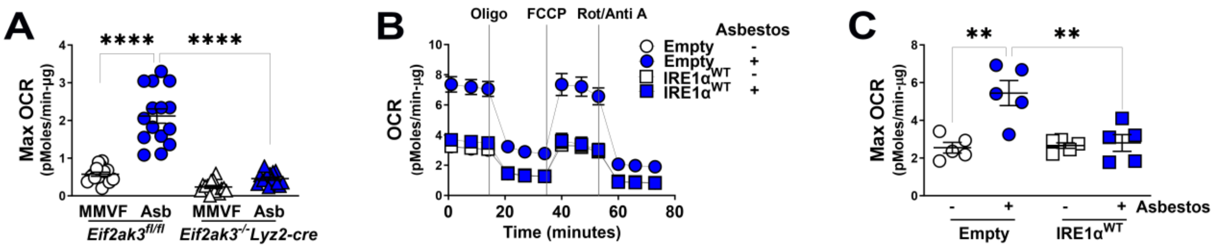

Supplemental Figure 3. Asbestos induced PERK-mediated metabolic reprogramming in lung macrophages. *Eif2ak3*$^{fl/fl}$ *and Eif2ak3*$^{-/-}$*Lyz2-cre* mice were exposed to MMVF or asbestos. **A** Seahorse assay performed for OCR measurement in BAL cells isolated at 21 days (*n*=3). Macrophages were transfected with empty or IRE1α^{WT} vectors without and with asbestos exposure. Seahorse assay performed for **B** OCR kinetics in macrophages (*n*=5) and **C** Max OCR in macrophages (*n*=5). Data shown as mean ± SEM. One-way ANOVA with Tukey's post hoc comparison. **$p \leq 0.01$ and ****$p \leq 0.0001$.

Supplemental Figure 4

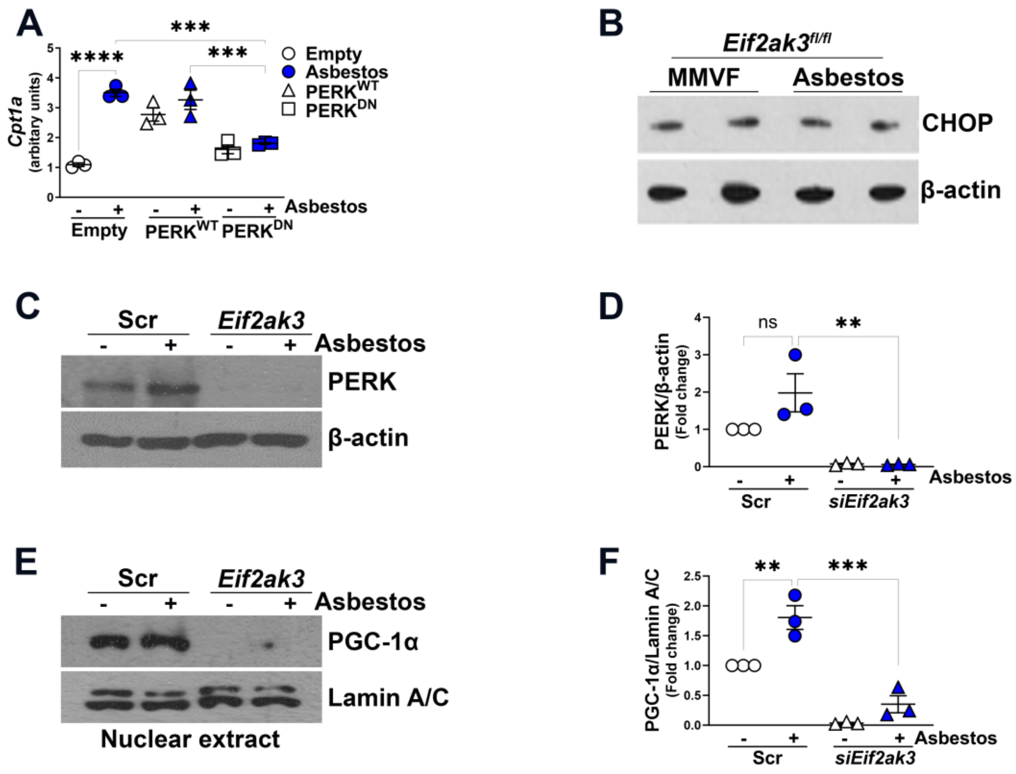

Supplemental Figure 4. PERK regulates *Cpt1a* and PGC-1α. Macrophages were transfected with empty, $PERK^{WT}$, or $PERK^{DN}$ without and with asbestos treatment. Total RNA was isolated and subjected to real-time PCR. **A** *Cpt1a* mRNA expression ($n = 3$). *Eif2ak3*$^{fl/fl}$ mice were exposed to MMVF or asbestos. **B** BAL was performed on day 21 and macrophages were subjected to immunoblot analysis of CHOP and β-actin (n=2). Macrophages were transfected with scramble or *Eif2ak3* siRNA and exposed to vehicle or asbestos. **C** Immunoblot analysis and **D** quantification of PERK and β-actin (n=3). **E** Immunoblot analysis and **F** quantification of PGC-1α and Lamin A/C (n=3). Data shown as mean ± SEM. One-way ANOVA with Tukey's post hoc comparison. $^{**}p \leq 0.01$, $^{***}p \leq 0.001$, and $^{****}p \leq 0.0001$.

Supplemental Figure 5

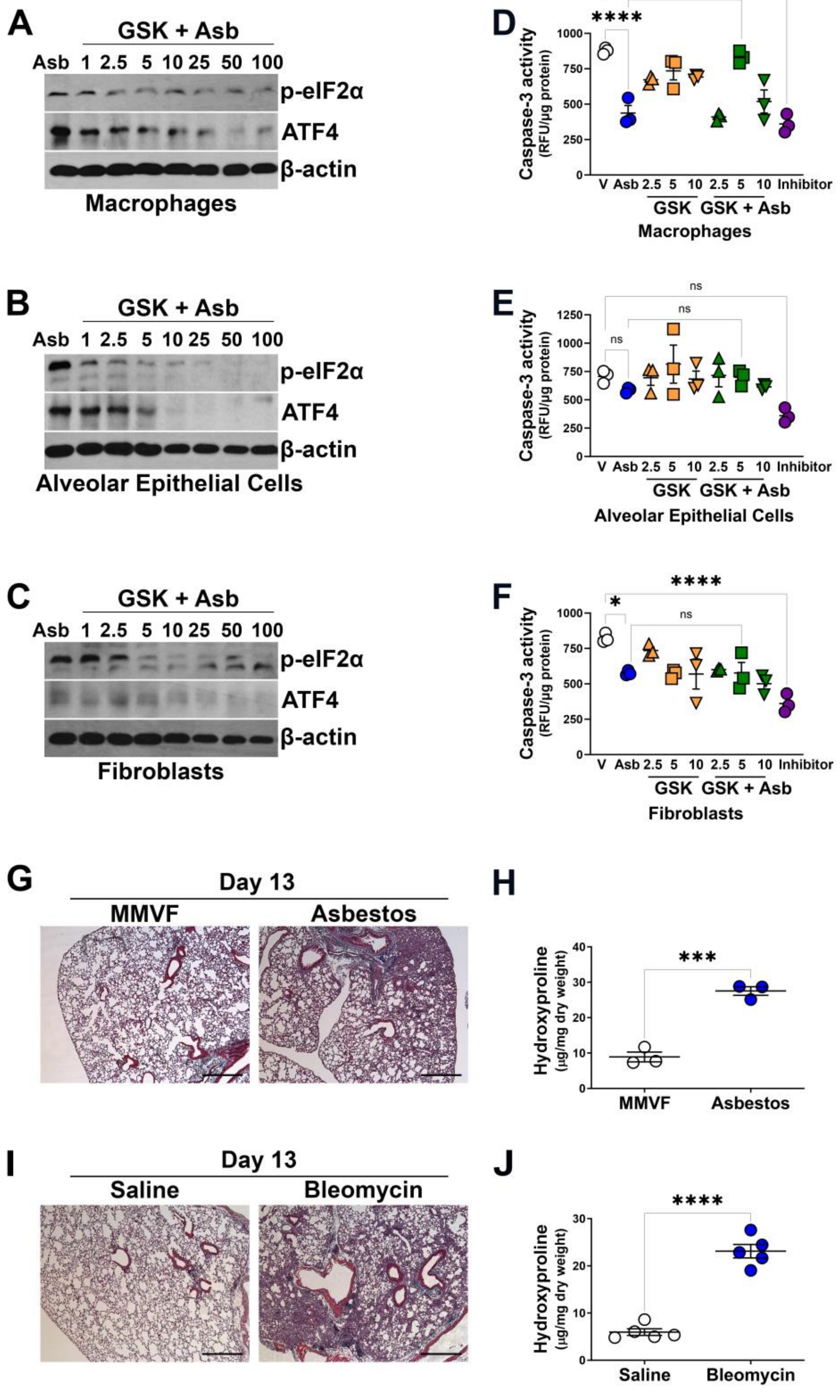

Supplemental Figure 5. PERK inhibition does not alter apoptosis. Immunoblot analysis of **A** macrophages, **B** AECs, and **C** fibroblasts pre-treated with GSK2656157 (1,

2.5, 5, 10, 25, 50 or 100μM) followed by exposure to asbestos (*n*=3). Caspase-3 activity in **D** macrophages, **E** AECs, and **F** fibroblasts treated with vehicle (V) or GSK2656157 and exposed to asbestos (*n*=3). A capase-3 inhibitor was used as a control. WT mice were exposed to MMVF or asbestos for 13 days and lungs were excised. **G** Masson's trichrome staining, scale bars at 500 μm, and **H** hydroxyproline was determined in lung tissues (*n*=3). WT mice were exposed to saline or bleomycin for 13 days and lungs were excised. **I** Masson's trichrome staining, scale bars at 500 μm, and **J** hydroxyproline was determined in lung tissues (*n*=5). Data shown as mean ± SEM. One-way ANOVA with Tukey's post hoc comparison in D-F. Two-tailed student's *t*-test in H and J. $^{*}p \leq 0.05$, $^{***}p \leq 0.001$, and $^{****}p \leq 0.0001$.

Supplemental Figure 6

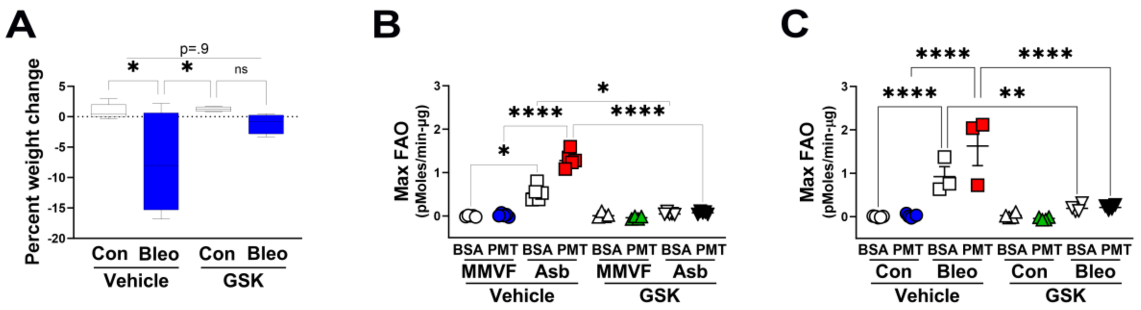

Supplemental Figure 6. PERK inhibition reduced FAO in macrophages from asbestos and bleomycin-injured mice. WT mice were exposed to control or bleomycin. GSK2656157 (30 mg/kg) or vehicle was administered daily beginning 13 days after exposure until day 21. **A** Percent change in body weight (*n*=5). **B** WT mice were exposed to MMVF or asbestos. GSK2656157 (30 mg/kg) or vehicle was administered daily beginning 13 days after exposure until day 21. Fatty acid oxidation (FAO) of lung macrophages was measured by OCR on the Seahorse XF96 bioanalyzer. Maximum FAO in lung macrophages (*n*=5). **C** Maximum FAO in lung macrophages from control or bleomycin exposed mice administered vehicle or GSK2656157 as detailed above (*n*=3-5). Data shown as mean ± SEM. One-way ANOVA with Tukey's post hoc comparison. *$p \leq 0.05$, **$p \leq 0.01$, and ****$p \leq 0.0001$.

Supplemental Figure 7

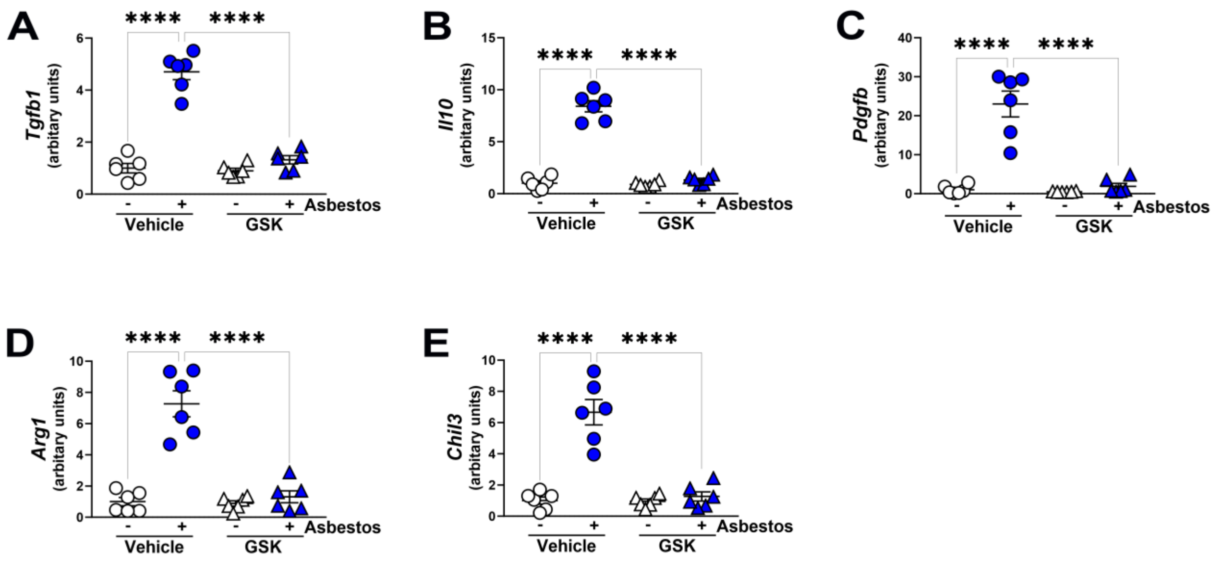

Supplemental Figure 7. PERK inhibition alters profibrotic gene expression. Macrophage were treated with vehicle or GSK2656157 (5 µM) and exposed to asbestos. Gene expression for **A** *Tgfb1* (*n*=6), **B** *Il10* (*n*=6), **C** *Pdgfb* (*n*=6), **D** *Arg1* (*n*=6), and **E** *Chil3* (*n*=6). Data shown as mean ± SEM. One-way ANOVA with Tukey's post hoc comparison. ****$p \leq 0.0001$.

References

1. Murthy S, Ryan A, He C, Mallampalli RK, and Carter AB. Rac1-mediated mitochondrial H2O2 generation regulates MMP-9 gene expression in macrophages via inhibition of SP-1 and AP-1. *J Biol Chem.* 2010;285(32):25062-73.
2. Carter AB, Knudtson KL, Monick MM, and Hunninghake GW. The p38 mitogen-activated protein kinase is required for NF-kappaB-dependent gene expression. The role of TATA-binding protein (TBP). *J Biol Chem.* 1999;274(43):30858-63.
3. He C, Ryan AJ, Murthy S, and Carter AB. Accelerated development of pulmonary fibrosis via Cu,Zn-superoxide dismutase-induced alternative activation of macrophages. *J Biol Chem.* 2013;288(28):20745-57.
4. Larson-Casey JL, Vaid M, Gu L, He C, Cai GQ, Ding Q, et al. Increased flux through the mevalonate pathway mediates fibrotic repair without injury. *J Clin Invest.* 2019;129(11):4962-78.
5. Gu L, Surolia R, Larson-Casey JL, He C, Davis D, Kang J, et al. Targeting Cpt1a-Bcl-2 interaction modulates apoptosis resistance and fibrotic remodeling. *Cell Death Differ.* 2022;29(1):118-32.
6. Pandey J, Larson-Casey JL, Patil MH, Joshi R, Jiang CS, Zhou Y, et al. NOX4-TIM23 interaction regulates NOX4 mitochondrial import and metabolic reprogramming. *J Biol Chem.* 2023;299(5):104695.
7. He C, Murthy S, McCormick ML, Spitz DR, Ryan AJ, and Carter AB. Mitochondrial Cu,Zn-superoxide dismutase mediates pulmonary fibrosis by augmenting H2O2 generation. *J Biol Chem.* 2011;286(17):15597-607.
