# Supplementary material for: The PERK/ATF4 pathway is required for metabolic reprogramming and progressive lung fibrosis
Source: JCI Insight. 2025 Apr 10;10(10):e189330. doi: 10.1172/jci.insight.189330 (PMC12128959; doi:10.1172/jci.insight.189330)

Full unedited gel for Figure 1A

p-PERK

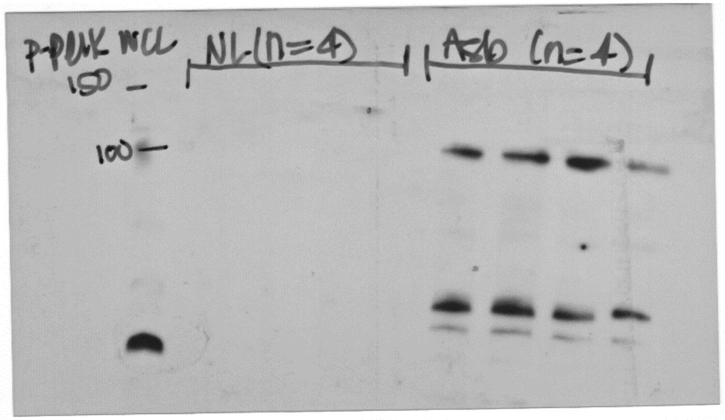

PERK

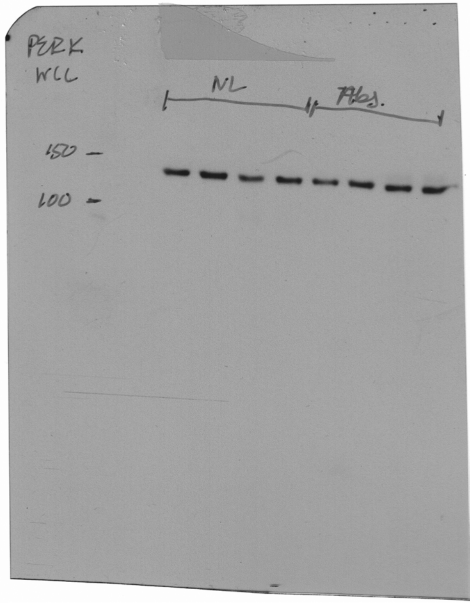

p-eIF2α

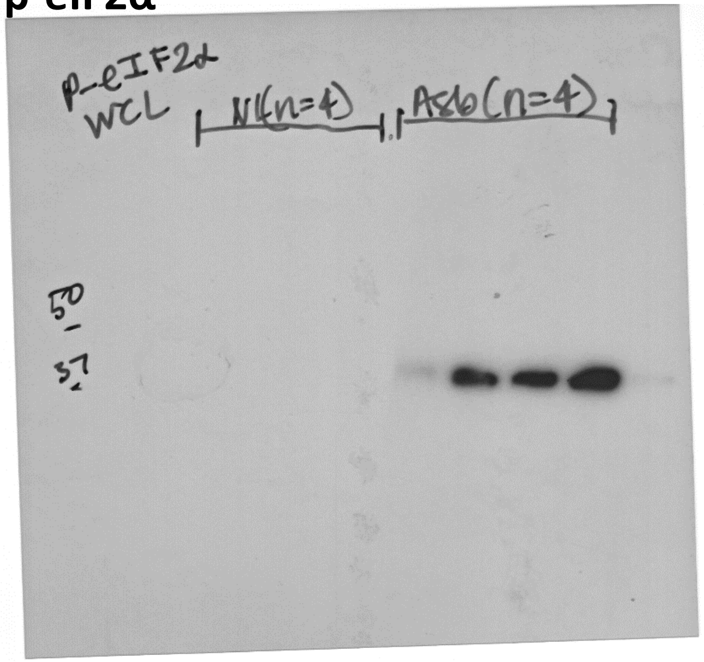

eIF2α

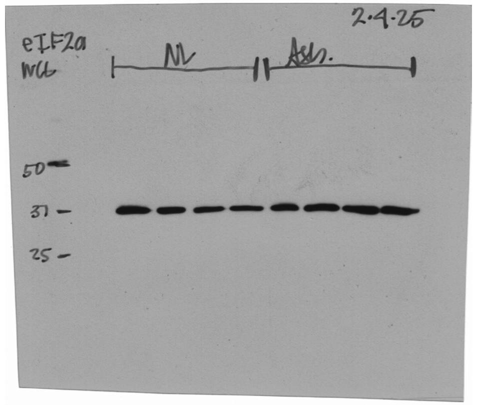

p-IRE1α

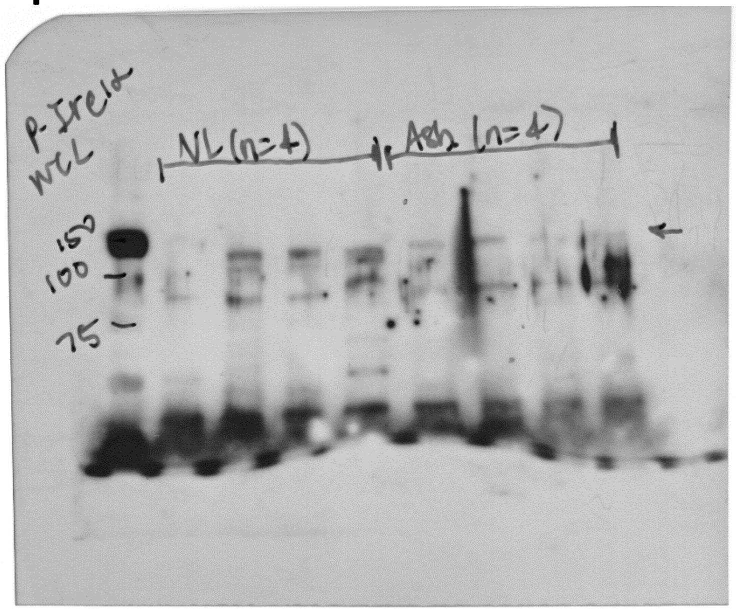

IRE1α

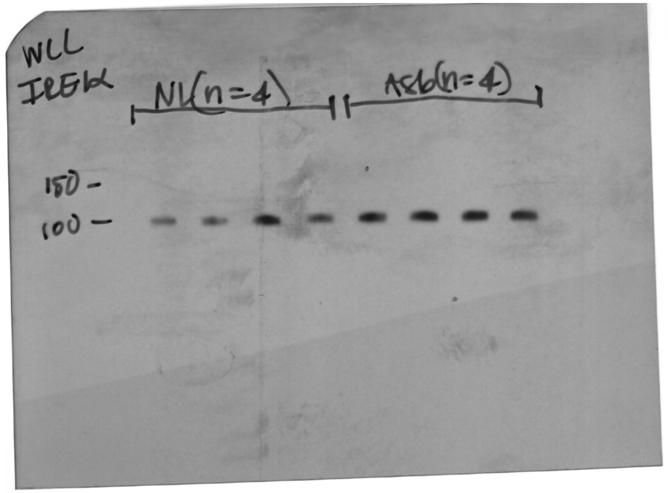

β-actin

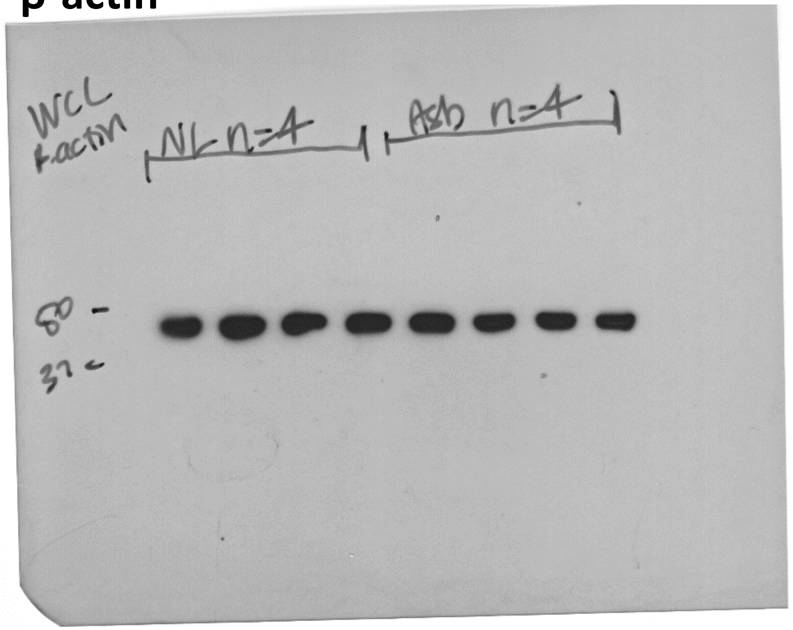

Full unedited gel for Figure 1E

p-PERK

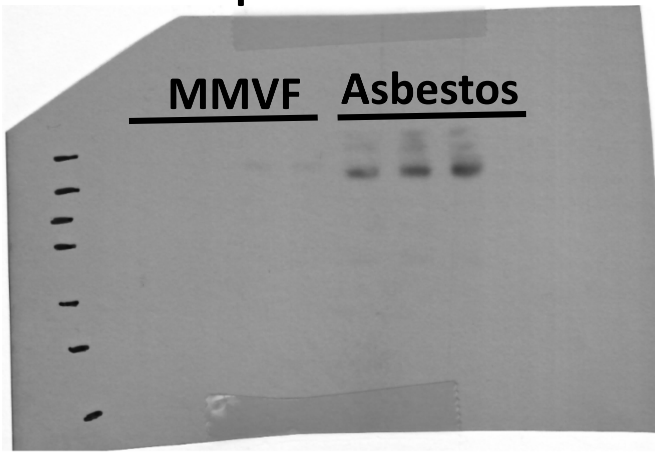

PERK

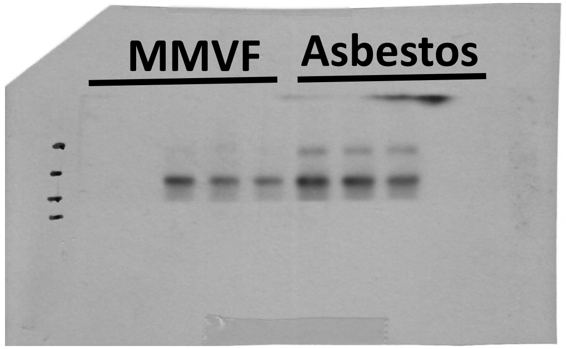

p-IRE1α

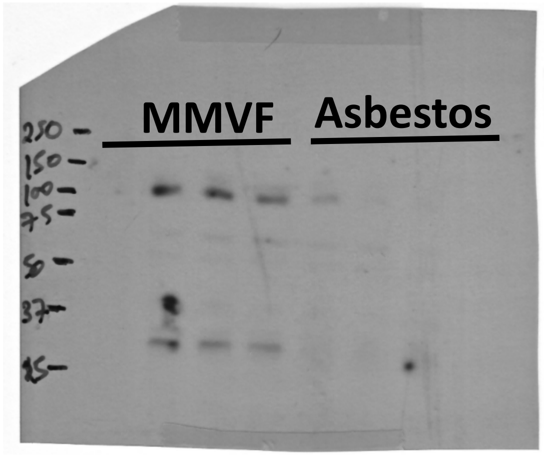

IRE1α

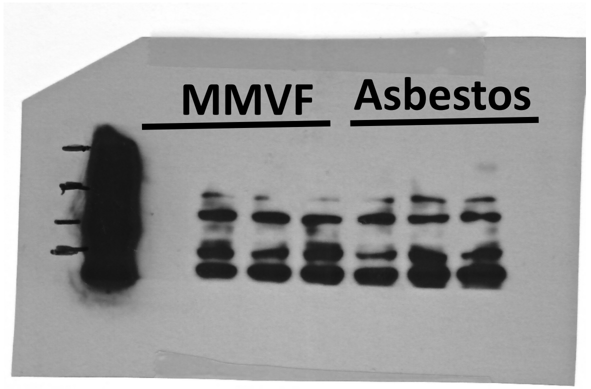

p-eIF2α

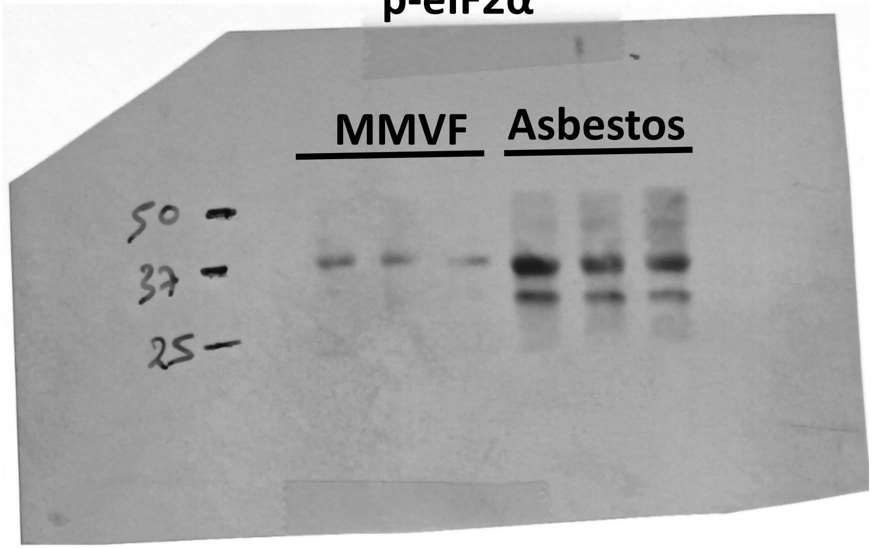

eIF2α

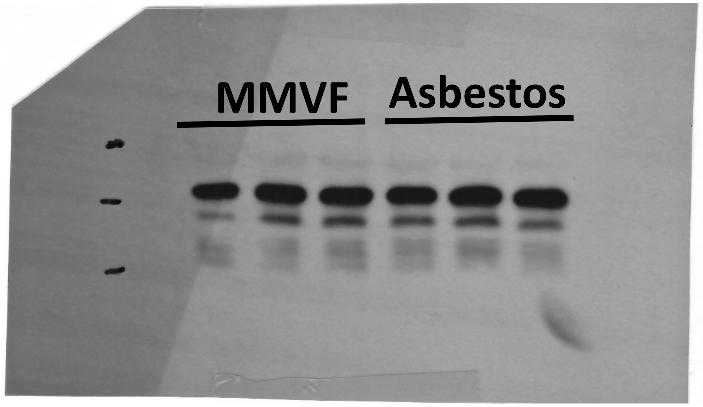

β-actin

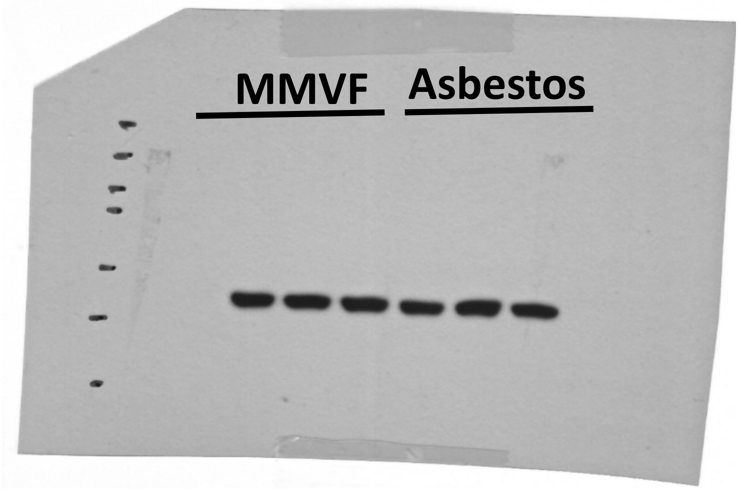

Full unedited gel for Figure 1I

BIP

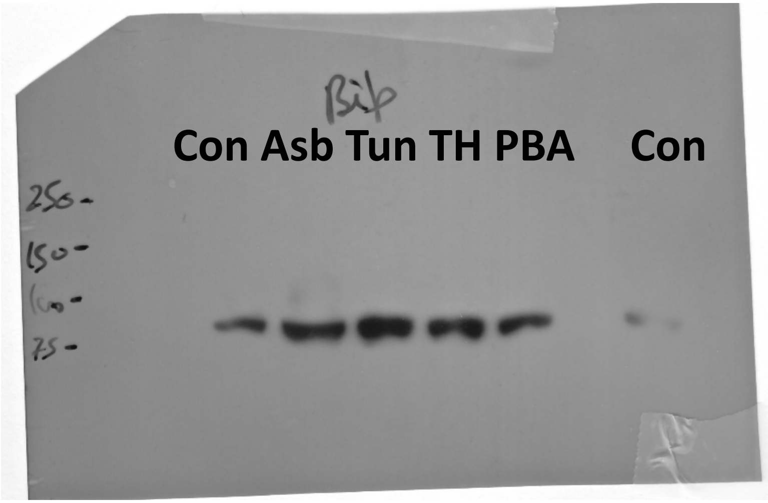

p-PERK

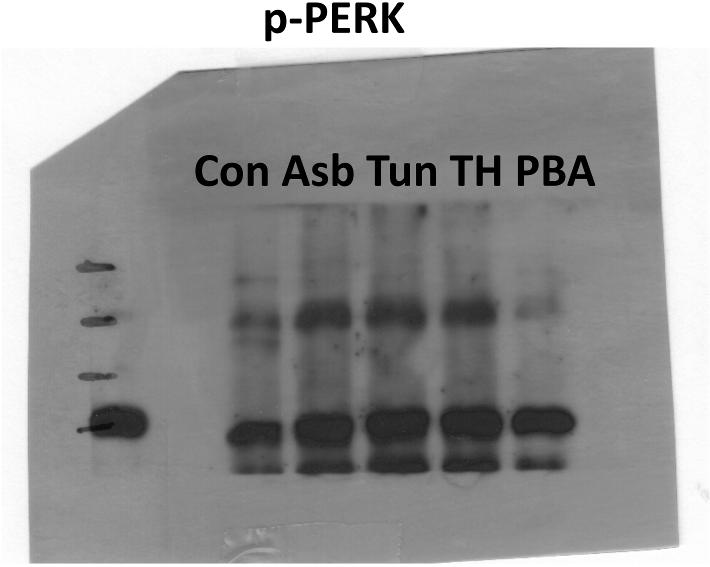

PERK

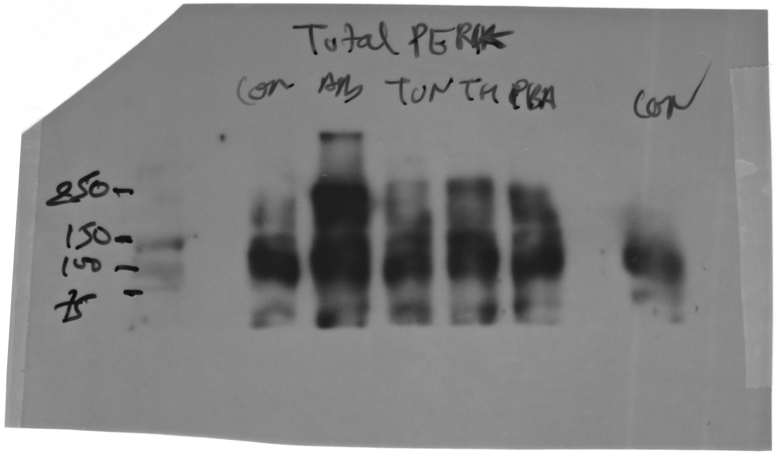

p-eIF2α

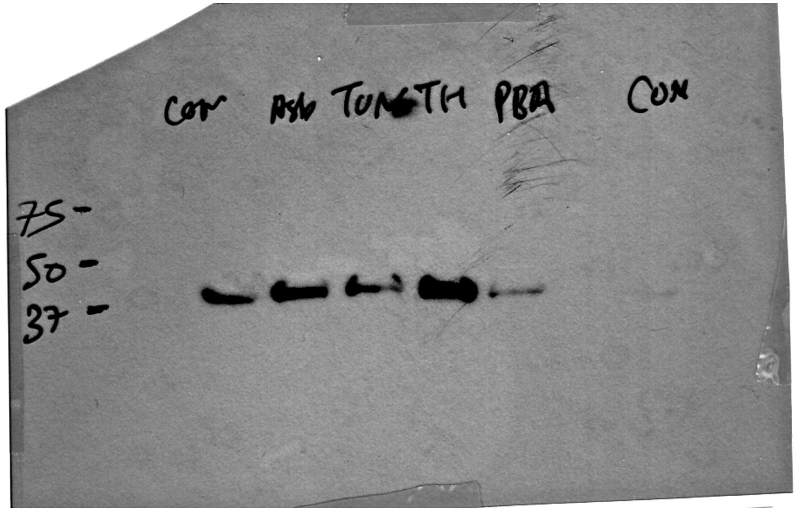

eIF2α

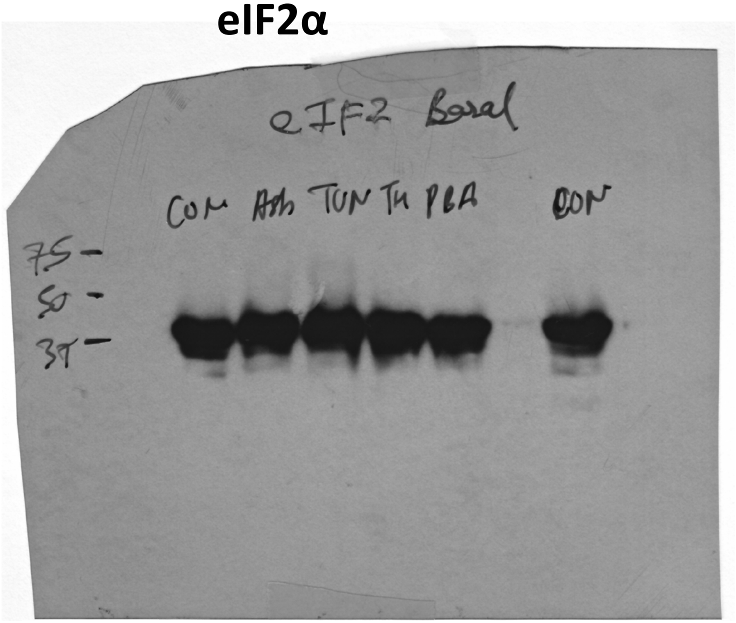

β-actin

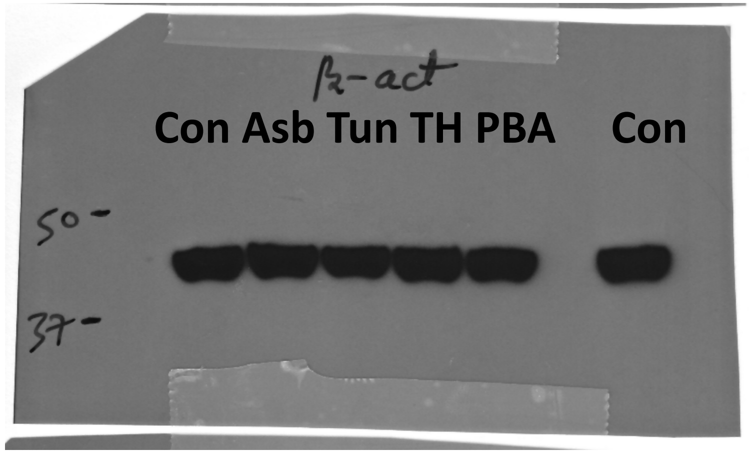

Full unedited gel for Figure 2C Inset

PERK

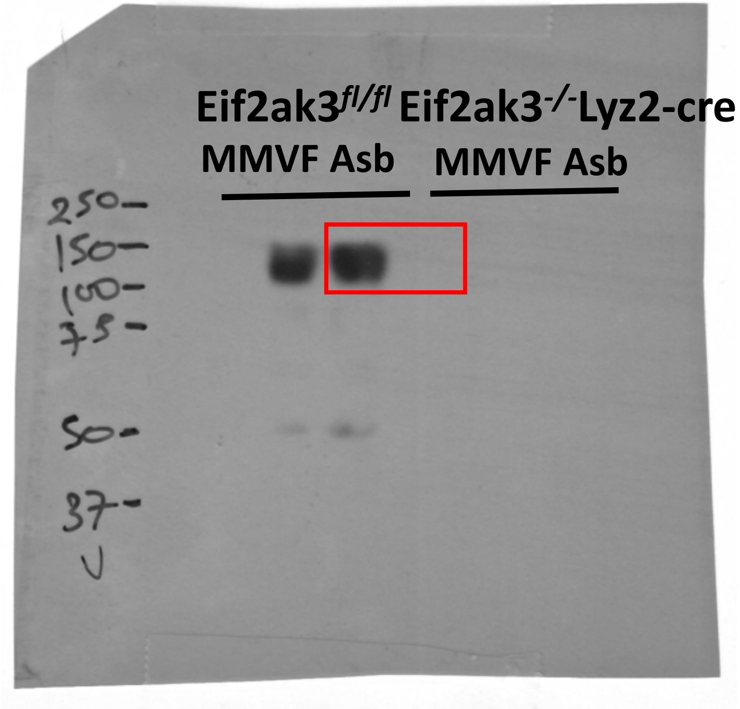

β-actin

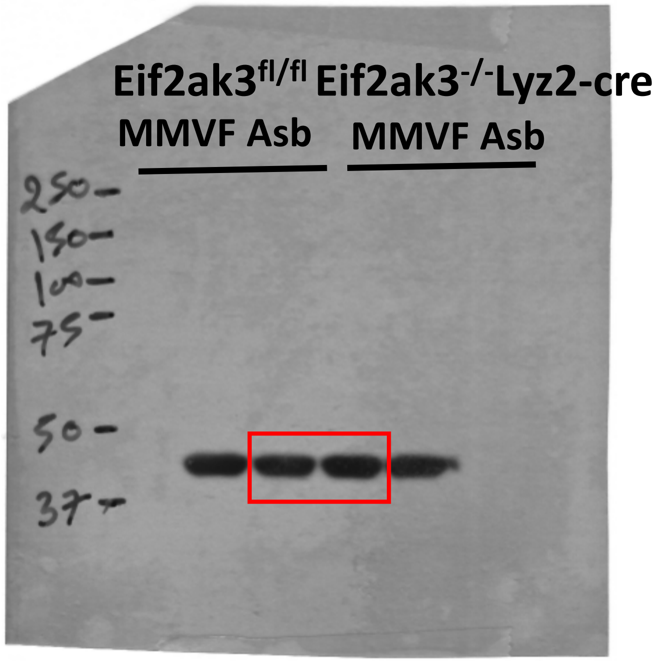

Full unedited gel for Figure 4D

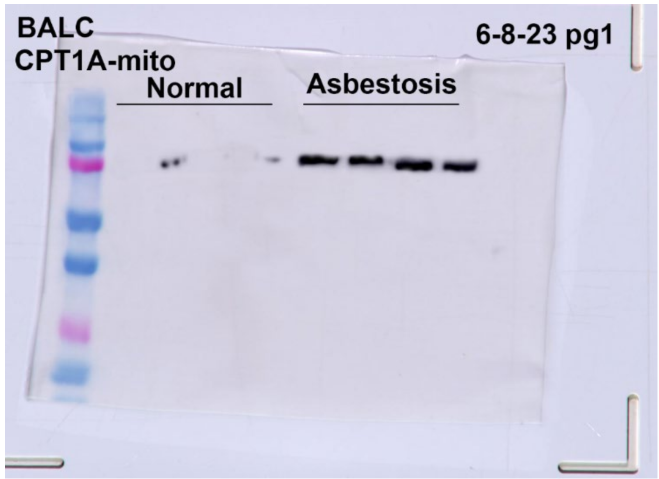

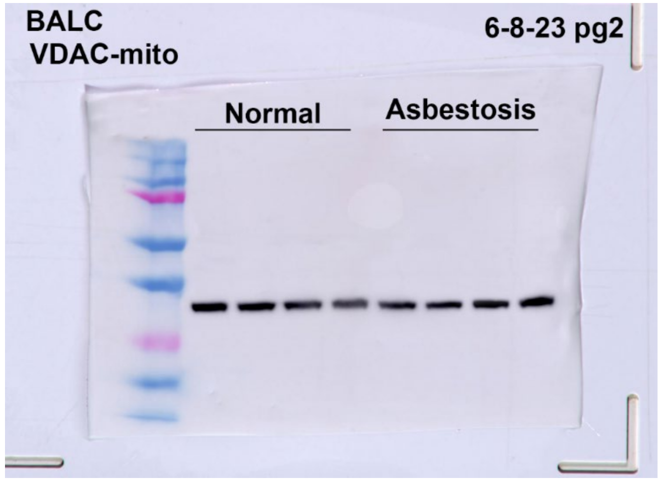

Full unedited gel for Figure 5B, Inset

PGC-1α

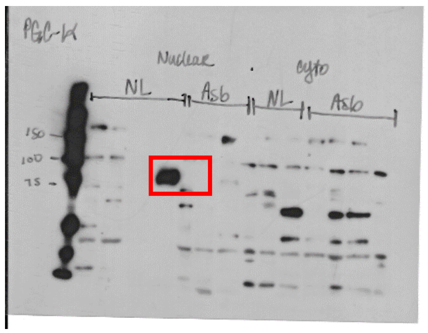

Lamin A/C

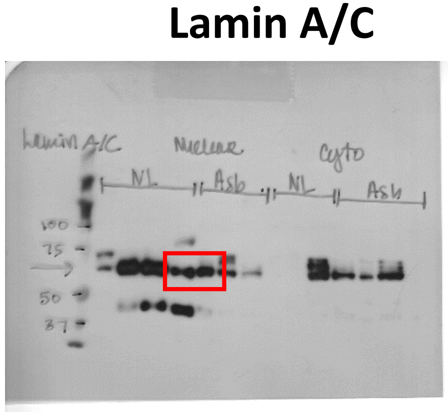

Full unedited gel for Figure 5H, Inset

ATF4

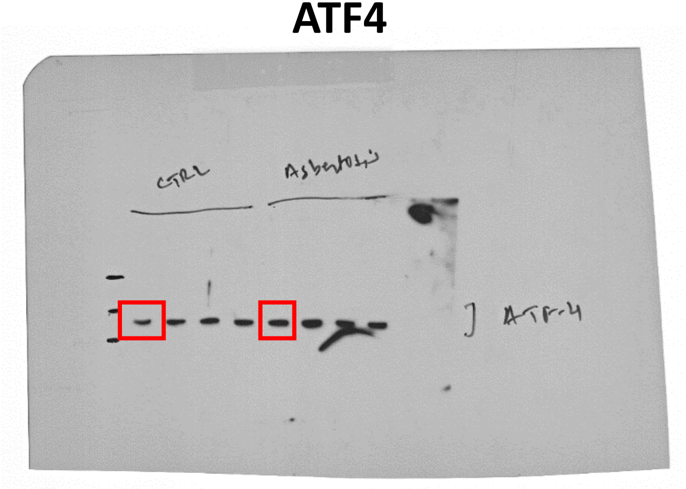

β-actin

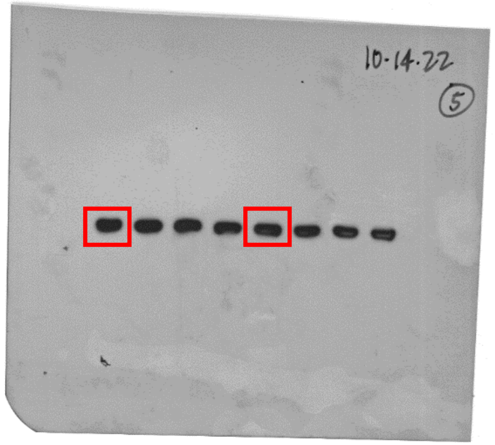

Full unedited gel for Figure 5I, Inset

ATF4

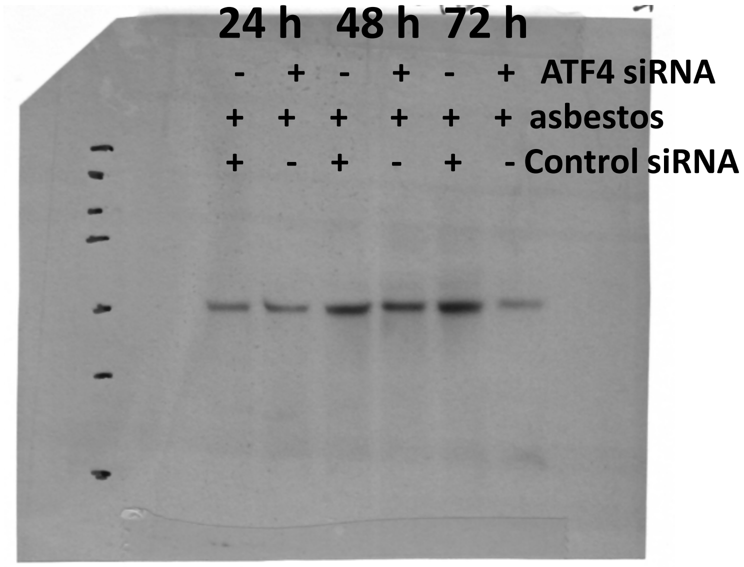

β-actin

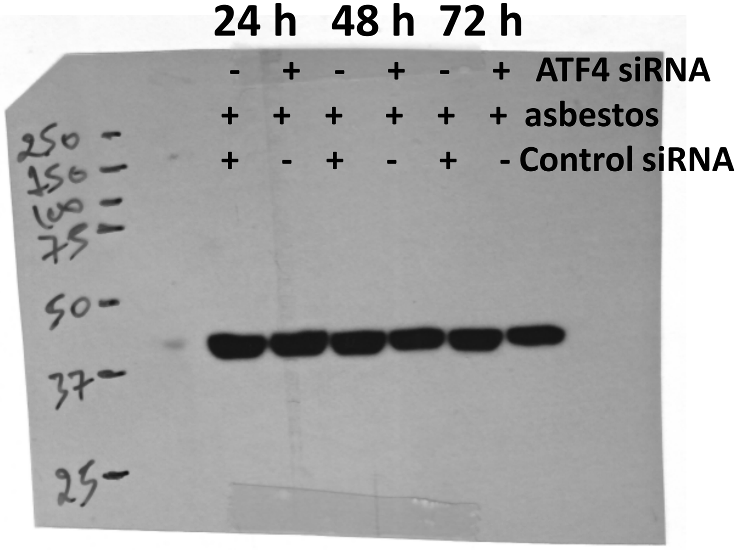

Full unedited gel for Supplemental Figure 1A

PERK

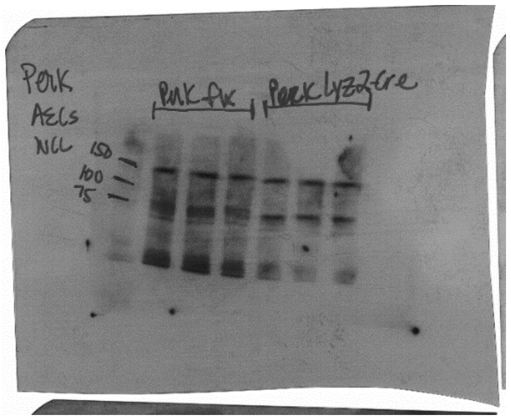

SPC

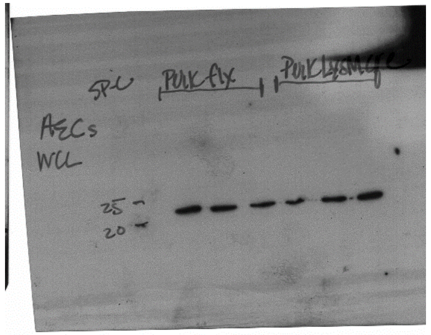

Full unedited gel for Supplemental Figure 4B

CHOP

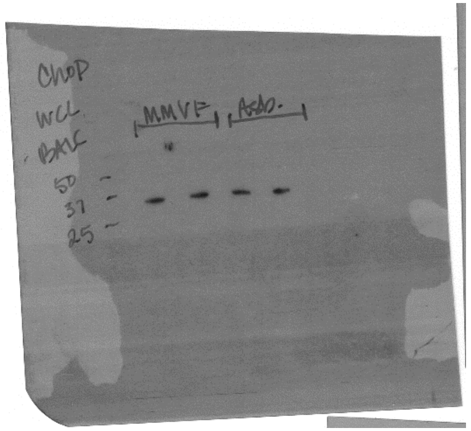

β-actin

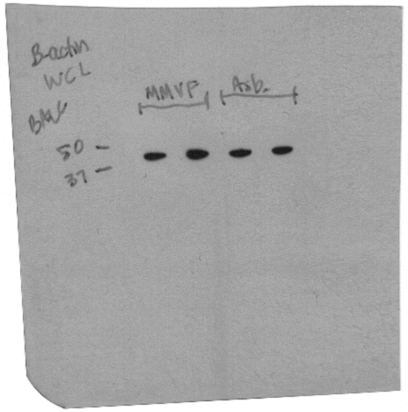

Full unedited gel for Supplemental Figure 4C

PERK

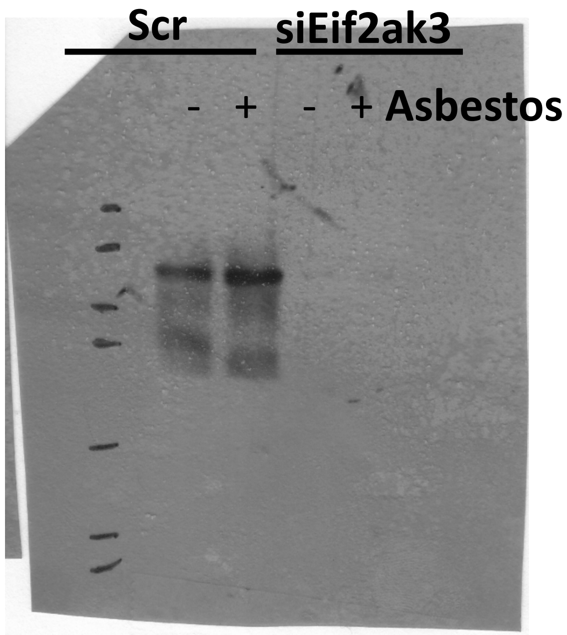

β-actin

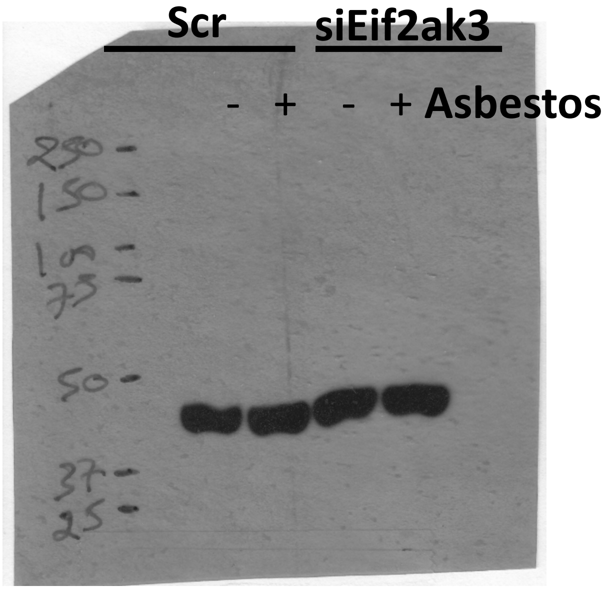

Full unedited gel for Supplemental Figure 4E

PGC-1α

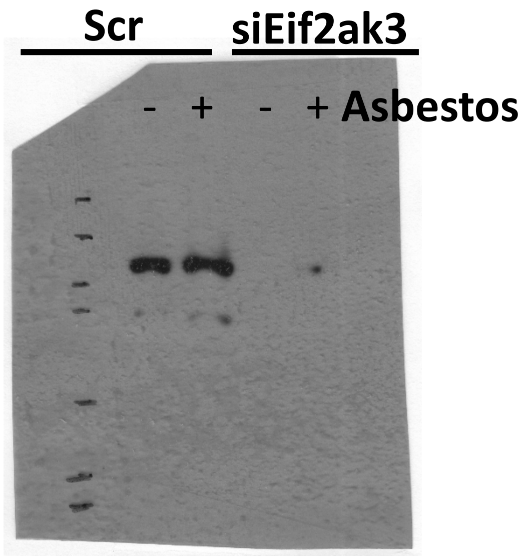

Lamin A/C

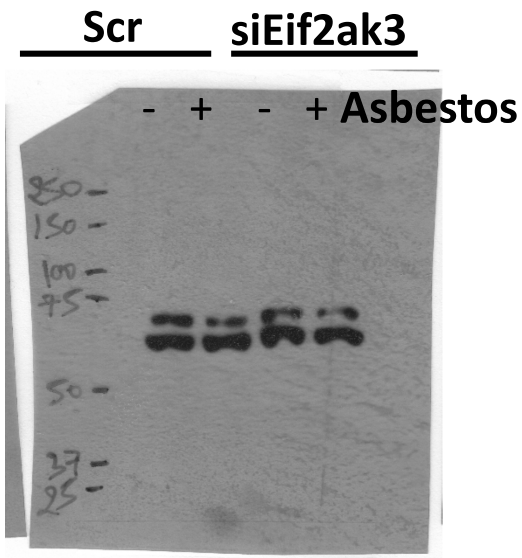

Full unedited gel for Supplemental Figure 5A

p-eIF2α

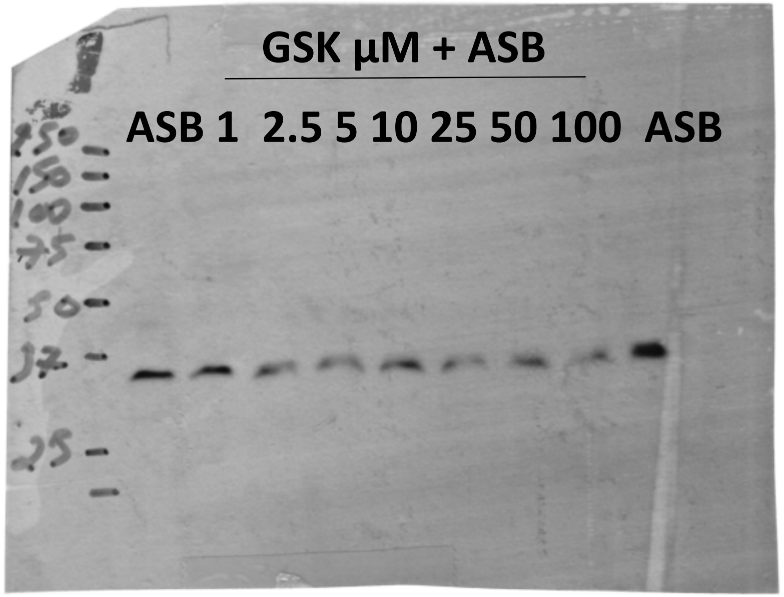

ATF4

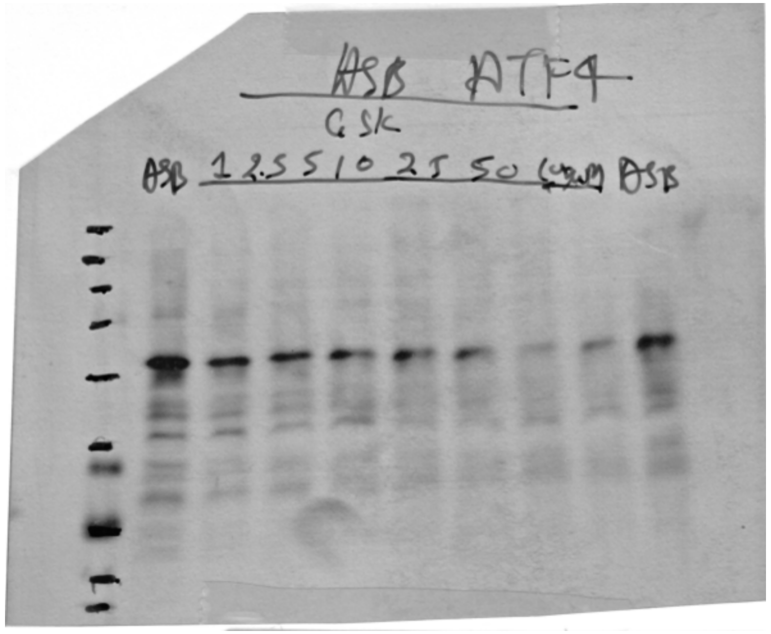

β-actin

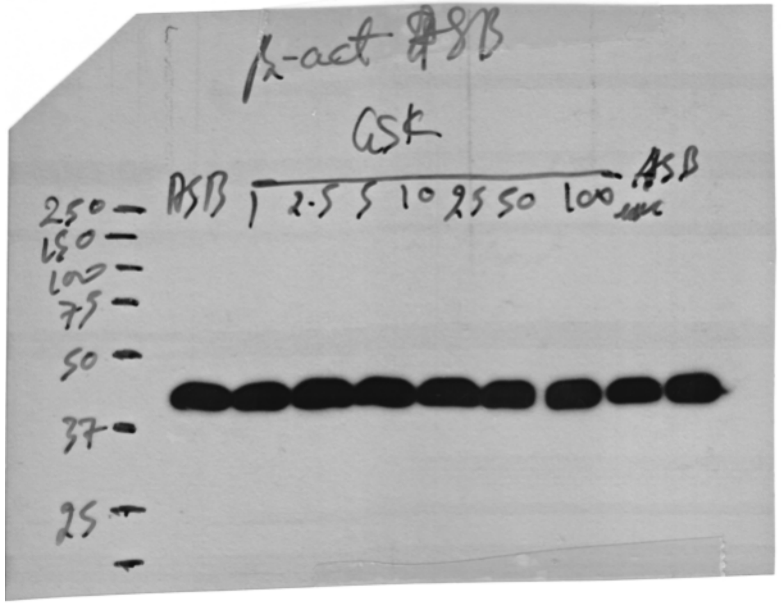

Full unedited gel for Supplemental Figure 5B

p-eIF2α

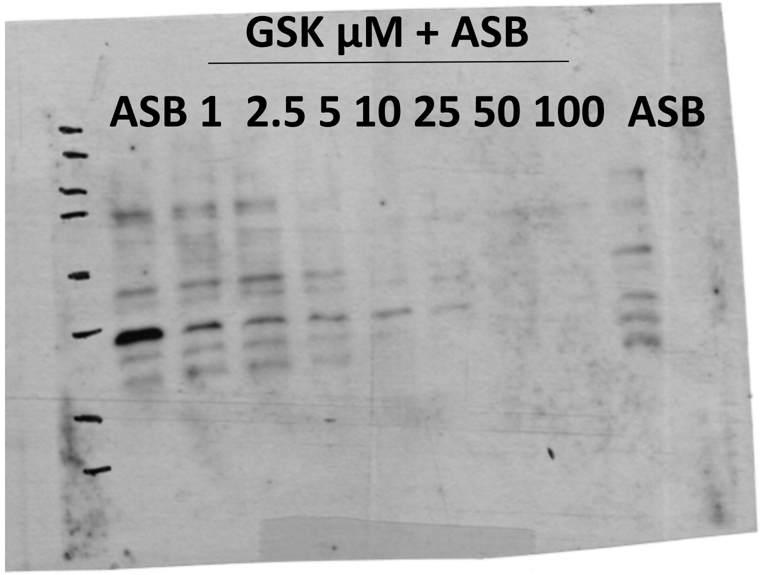

ATF4

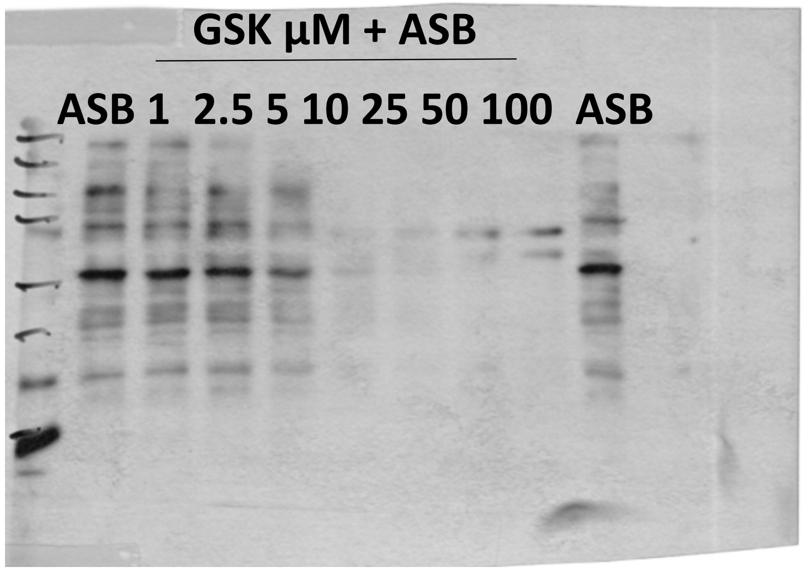

β -actin

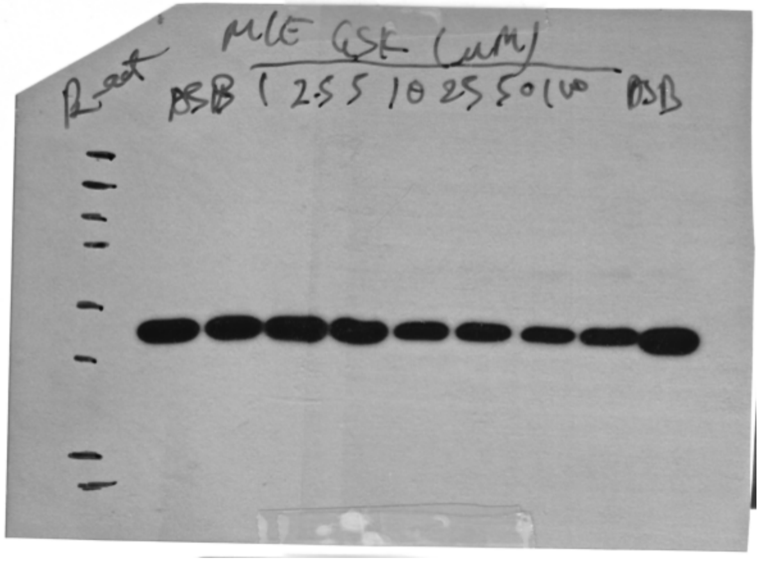

Full unedited gel for Supplemental Figure 5C

p-eIF2α

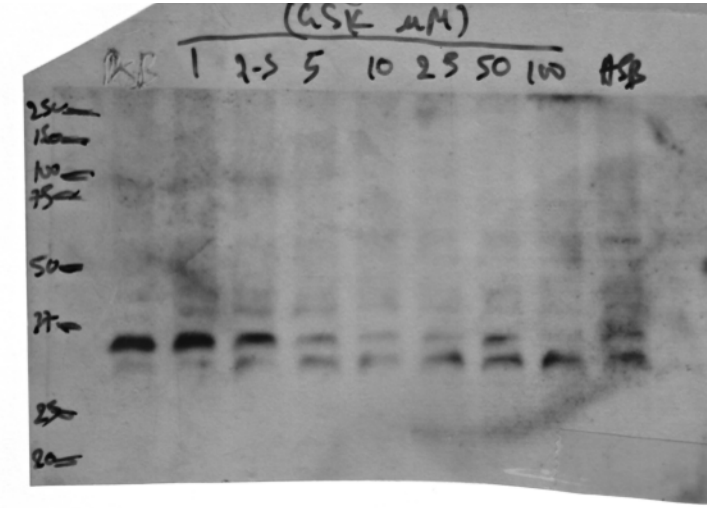

ATF4

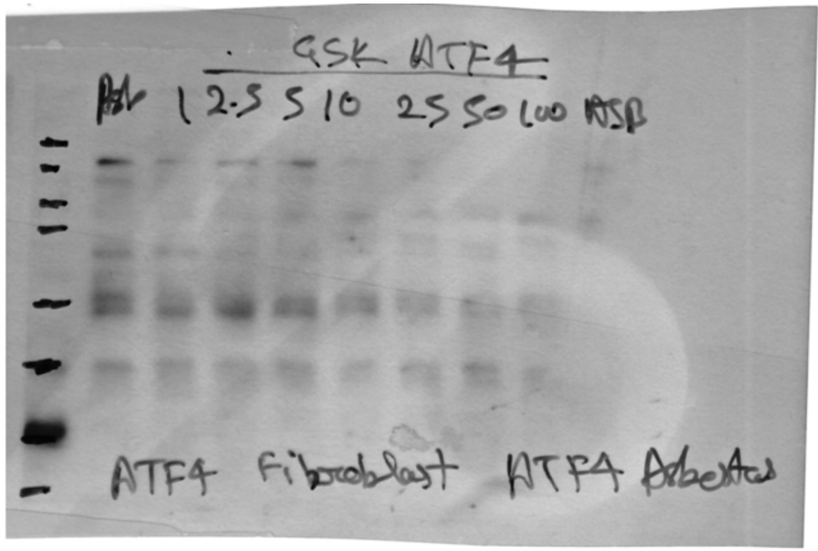

β -actin

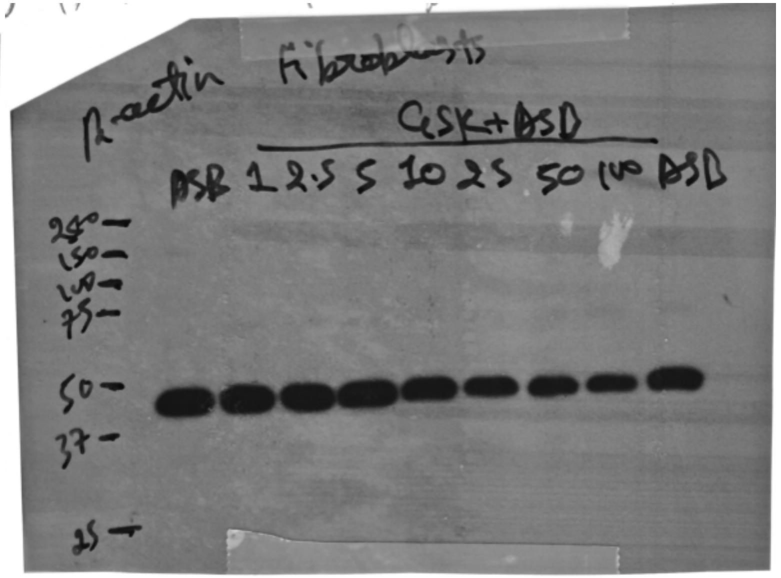

Supplement: Unedited blot and gel images [file jciinsight-10-189330-s020.pdf]
